# Phenotype Specific Analyses Reveal Distinct Regulatory Mechanism for Chronically Activated p53

**DOI:** 10.1371/journal.pgen.1005053

**Published:** 2015-03-19

**Authors:** Kristina Kirschner, Shamith A. Samarajiwa, Jonathan M. Cairns, Suraj Menon, Pedro A. Pérez-Mancera, Kosuke Tomimatsu, Camino Bermejo-Rodriguez, Yoko Ito, Tamir Chandra, Masako Narita, Scott K. Lyons, Andy G. Lynch, Hiroshi Kimura, Tetsuya Ohbayashi, Simon Tavaré, Masashi Narita

**Affiliations:** 1 Cancer Research UK Cambridge Institute, University of Cambridge, Li Ka Shing Centre, Cambridge, United Kingdom; 2 Graduate School of Bioscience and Biotechnology, Tokyo Institute of Technology, Yokohama, Japan; 3 Research Center for Bioscience and Technology, Tottori University, Yonago, Japan; Imperial College Faculty of Medicine, UNITED KINGDOM

## Abstract

The downstream functions of the DNA binding tumor suppressor p53 vary depending on the cellular context, and persistent p53 activation has recently been implicated in tumor suppression and senescence. However, genome-wide information about p53-target gene regulation has been derived mostly from acute genotoxic conditions. Using ChIP-seq and expression data, we have found distinct p53 binding profiles between acutely activated (through DNA damage) and chronically activated (in senescent or pro-apoptotic conditions) p53. Compared to the classical ‘acute’ p53 binding profile, ‘chronic’ p53 peaks were closely associated with CpG-islands. Furthermore, the chronic CpG-island binding of p53 conferred distinct expression patterns between senescent and pro-apoptotic conditions. Using the p53 targets seen in the chronic conditions together with external high-throughput datasets, we have built p53 networks that revealed extensive self-regulatory ‘p53 hubs’ where p53 and many p53 targets can physically interact with each other. Integrating these results with public clinical datasets identified the cancer-associated lipogenic enzyme, *SCD*, which we found to be directly repressed by p53 through the CpG-island promoter, providing a mechanistic link between p53 and the ‘lipogenic phenotype’, a hallmark of cancer. Our data reveal distinct phenotype associations of chronic p53 targets that underlie specific gene regulatory mechanisms.

## Author Summary

The p53 transcription factor is a frequently mutated tumour suppressor that contributes to repairing or eliminating damaged cells. Levels of p53 are typically regulated through its stability; it is constantly produced and degraded, so that upon stress, p53 is up-regulated quickly. This acutely induced p53 has been used as a major model system for studying genome-wide p53 targets. However, emerging evidence suggests that persistently activated p53 is involved in cancer-associated phenotypes, such as cellular senescence. We investigate genome-wide gene regulation by acutely induced p53 through DNA damage as well as chronically activated p53 in oncogene-induced senescence and pro-apoptotic states. Interestingly, acute and chronic p53 DNA binding profiles are highly distinctive, the latter being preferentially associated with larger and relatively open promoters called CpG islands. Furthermore, our integrative analyses of both p53-dependent gene expression and p53-binding genomic DNA profiles reveal that p53 and many of its targets in chronic conditions form extensive self-regulatory hubs, where they can physically interact. The data not only substantially extend the list of direct p53 targets but highlight unique gene regulation by chronic p53. Finally we show that the cancer-associated lipogenic enzyme, *stearoyl-CoA desaturase*, is a *bona fide* p53-repressive target through its CpG island promoter in chronic conditions.

## Introduction

The TP53 (p53) tumor suppressor, a stress-responsive transcription factor (TF), is somatically mutated in more than 50% of human cancers, with a range between 10% and nearly 100% depending on the tumor type. Furthermore, germ line mutations of p53, in both humans and mice, predispose individuals to malignant tumor development [1,2]. p53 plays critical roles in the induction of cell death and cell cycle arrest in response to stress, including DNA damage, oncogenic stress, and metabolic stress. Hence p53 is implicated in a wide range of cellular processes, such as cell cycle checkpoint, apoptosis, senescence and quiescence [3–5]. Despite increasing knowledge about p53 target genes, however, it is not entirely clear which aspects of p53 function are attributable to each of these p53-associated phenotypes and its tumor suppressor activity [6].

p53 is typically regulated at the protein level through post-translational modification. In normal conditions, p53 is under the regulation of a strong negative feedback loop, where MDM2, a direct p53 target, serves as the E3 ubiquitin ligase, leading to the constant proteasomal degradation of p53 [7]. Thus p53 is highly unstable in non-stress conditions but upon stress induction, such as DNA damage, it can be rapidly stabilized through its dissociation from MDM2. However, whether or not the prevailing model of acute p53 induction represents the major program of p53’s tumor suppressive functions is under debate [8]. For example, studying whole body irradiated p53 inducible knock-in mice, Christophorou et al. showed that a late restoration of p53 function, rather than the usual acute p53-mediated pathological response, led to a reduced lymphoma burden [9]. In addition, Brady et al. recently showed that p53 differentially regulates specific transcriptional programs of the acute DNA damage response (DDR) and its more chronic tumor suppression functions through its use of different transactivation domains. Their data indicate a close correlation between p53 activities in driving tumor suppression and senescence [10]. Notably, senescence has been shown to be largely dependent on a persistent, rather than an acute, DDR [11]. Thus these studies suggest that the downstream effects of acutely activated p53 and p53-mediated tumor suppression may well be separable processes.

Several studies of p53 genomic binding profile have recently been published, revealing a number of new p53 targets, which include genes potentially associated with its tumor suppressor functions. An early study found p53 targets that potentially suppress metastasis [12]. A number of autophagy genes were recently identified as direct p53 targets and p53-induced autophagy was shown to be important for DNA damage-induced apoptosis and the anti-transformation activity of p53 [13]. In addition, in ES cells, p53 regulates self-renewal and pluripotency upon DNA damage [14], and early-differentiation p53 targets include many developmental transcription factors [15]. Currently, however, efforts at genome-wide p53 mapping have mostly been focused on acutely or dynamically activated p53. Thus comprehensive analyses of the persistent activities of p53, which may be more relevant to its tumor suppressor function, are still missing.

Here we show distinct regulatory mechanisms for p53-targets between acute and more persistent modes of p53 activation. In addition to the classical DDR, where p53 is acutely induced (‘acute’ p53), we have determined profiles of genome-wide p53 binding and p53-responsive genes in two distinct cellular conditions, where p53 is persistently activated (‘chronic’ p53) in normal human diploid fibroblasts (HDFs): oncogene-induced senescence (OIS); and transformed pro-apoptotic conditions. In contrast to acute p53, chronic p53 was closely associated with CpG island (CGI) type promoters. Although the binding profiles of p53 in the OIS and pro-apoptotic conditions were similar, the p53-responsive genes were distinct, suggesting that downstream gene regulation by chronic p53 is highly context dependent. Interestingly, our integrative p53 networks and pathway modeling, combined with external high-throughput datasets, suggest that p53 can be functionally and/or physically associated with many of its own targets, thus forming extensive self-regulatory p53 hubs in the chronic conditions examined in this study. Finally, together with external clinical datasets, our data reinforce the evidence for the anti-lipogenic functions for p53. Our study not only extends our knowledge of phenotype-associated gene regulation by p53, but also provides unique and widely useful resources for the targets of persistently activated p53.

## Results

### Phenotype-specific p53-responsive gene expression

**Fig 1:**
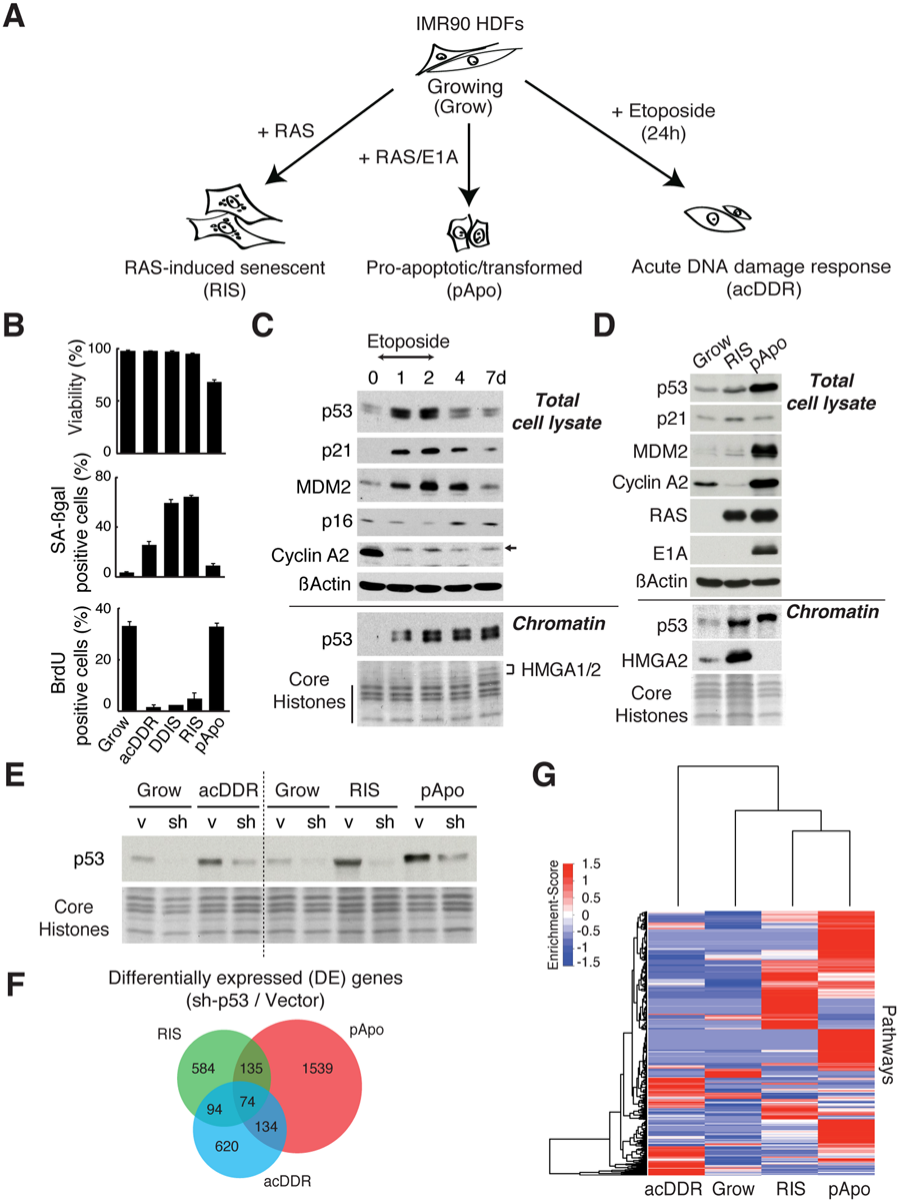
Phenotype-associated p53-responsive gene expression in IMR90 cells. **(A)** Schematic of the p53-associated phenotypes. **(B)** Cell viability, senescence-associated ß-galactosidase activity (SA-ß-gal), and BrdU incorporation (mean ± SEM; n = 3) were measured for each condition as indicated in (A). In addition, DNA damage-induced senescence (DDIS) was included for comparison: cells were treated with etoposide (100 μM) for two days, and maintained for an additional five days in drug-free media. **(C, D)** Immunoblot analyses for the proteins indicated for total lysates and chromatin fractions from the cells as labeled. Cyclin A2, a cell cycle marker; HMGA proteins, senescence markers. d1 and d7 correspond to acDDR and DDIS, respectively (C). Core histones (C, D) and HMGA proteins (C) were stained with Coomassie blue. The arrow indicates non-specific bands (the Cyclin A2 blot in (C)). **(E)** Immunoblot analysis in the indicated cells for chromatin fractions for p53. sh and v, sh-p53#1 and corresponding lentiviral vector (a miR30 design), respectively. For acDDR, sh-p53 was introduced first for at least 5 days before administration of etoposide. For RIS and pApo, sh-p53 was introduced after the phenotype establishment. Core histones were stained with Coomassie blue. **(F)** Venn diagram showing the numbers of differentially expressed (DE) genes upon p53 depletion with lentivirus-mediated RNAi (sh-p53#1) compared to vector, in the indicated conditions. **(G)** Pathway heatmap for differentially expressed genes upon p53 depletion.

To gain a comprehensive understanding of p53 biology, we established phenotypes that are associated with p53 either acutely activated by DNA damage or persistently activated by oncogenic stress in a single cell type (IMR90 HDFs) (Fig. 1A). During the acute DNA damage response (acDDR) phase induced by etoposide treatment (d1), the cells were viable and had stopped proliferating but were not yet fully senescent, whereas most cells became senescent seven days after etoposide treatment (Fig. 1B-1C). Of note, although acDDR cells showed a modest increase in senescence-associated ß-galactosidase activity (Fig. 1B), it was not accompanied by up-regulation of other functional markers of senescence, such as HMGA proteins and p16 (Fig. 1C). As expected, p53 was transiently stabilized in the acDDR phase with a parallel up-regulation of p53 targets, such as p21 and MDM2, in total cell lysates (Fig. 1C). Interestingly, in chromatin-enriched fractions, p53 levels were comparable between the acute (d1) and senescence phases (d7). This is perhaps in part due to the enlarged cellular phenotype of senescent cells, the p53 level then being more diluted in total cell lysates of senescent cells.

To establish the conditions for the sustained activation of p53, we used the well-established models of oncogenic stress [16]. Ectopic oncogenic HRAS^G12V^ induces senescence (RAS-induced senescence, RIS), a state of irreversible cell cycle arrest, where p53 plays a major role [16]. In contrast, E1A, the ‘immortalizing’ adenoviral oncoprotein, transforms HDFs when used in combination with oncogenic HRAS^G12V^. At the same time, E1A stabilizes p53 and thereby sensitizes cells to apoptosis (Figs. 1A, 1D, and S1A) [17]. Thus E1A/RAS-expressing cells are highly proliferative, yet sensitive to apoptosis due to sustained p53 activation (here we call this condition ‘pro-apoptotic’, pApo). In both cases, a stable accumulation of p53 was readily detectable in chromatin fractions without additional stimuli (Fig. 1D), and again the elevated levels of p53, particularly in the RIS condition, were more clearly detected in chromatin fractions than in total lysates (Fig. 1D). These data suggest that comparable amounts of p53 can be responsible for the distinct phenotypes.

Having established highly distinct p53-associated phenotypes—acDDR, RIS, and pApo—we performed microarray analysis, with and without sh-p53, for each condition using a miR30 RNAi design in the lentiviral backbone [18]. To reduce secondary effects of p53 knockdown, we introduced sh-p53 after each phenotype was established in the chronic conditions. The efficiency of p53 knockdown was confirmed in the chromatin fractions (Fig. 1E). The set of differentially expressed (DE) genes upon sh-p53 introduction in each phenotype differed greatly between all conditions, with only a small number of well-characterized p53 targets in common (Figs. 1F and S1B, and S1 Table). Regulation of three representative core p53 targets was validated using a different sh-p53 (S1C Fig). Pathway analyses of DE gene sets confirmed distinct transcriptional signatures in each phenotype (Figs. 1G and S1D), indicating that p53 can, directly or indirectly, regulate gene sets unique in terms of both their context and phenotype, i.e. in either the ‘acute’ or ‘chronic’ p53 condition, and the RIS or pApo condition.

### Distinct genomic binding profiles of p53 between the acute and chronic conditions

**Fig 2:**
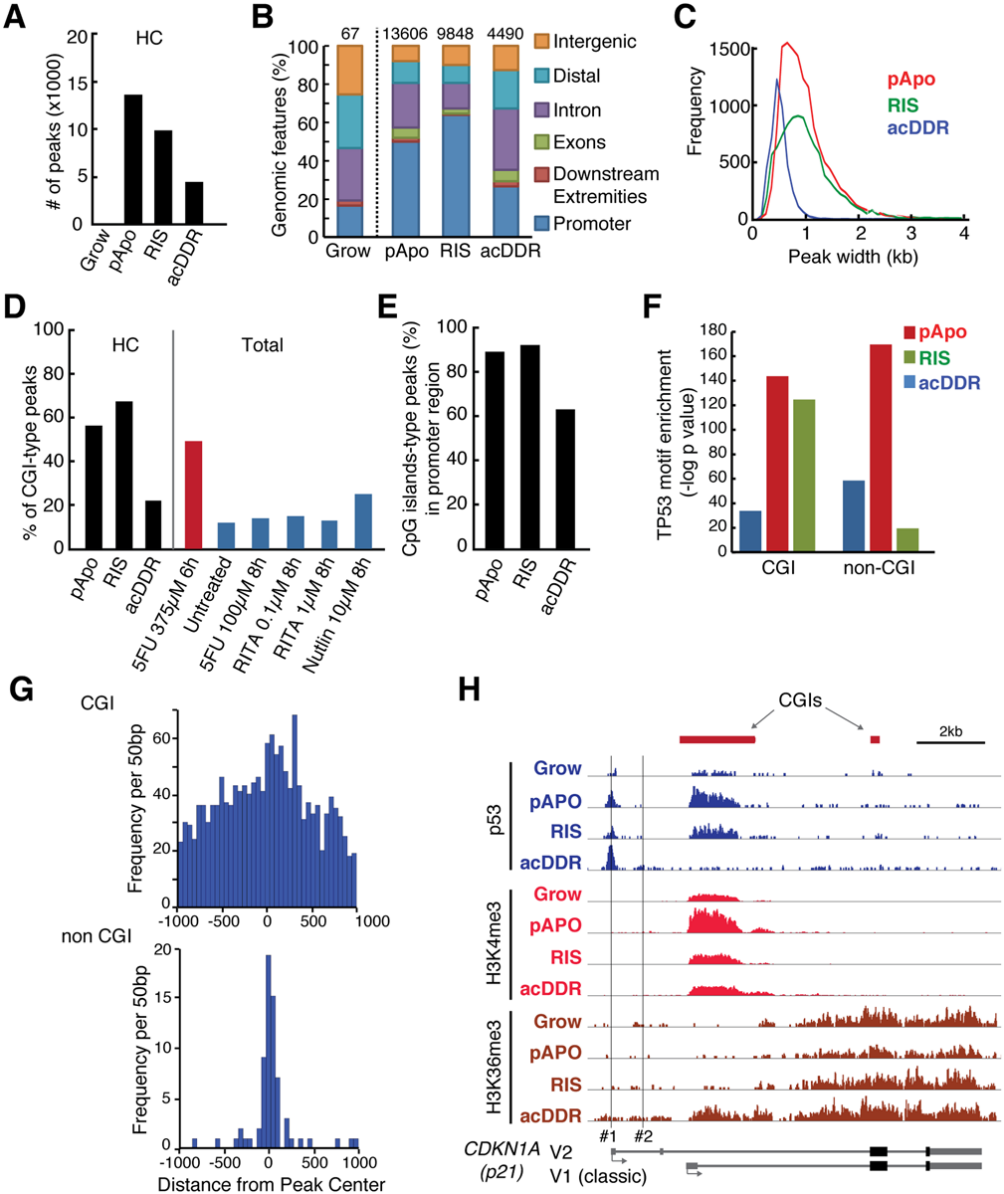
‘Chronic’ p53 preferably associates with CpG islands, whereas ‘acute’ p53 exhibits a diverse genomic distribution. **(A, B)** Number (A) and genomic features (B) of high confidence (HC) p53 ChIP-seq peaks from the indicated conditions. The number of HC peaks from (A) is also shown in (B). **(C)** Peak-width distribution in each condition. **(D)** Proportion of peaks associated with CpG islands (CGIs) to our HC peaks (black bars) and total peaks from external datasets: red bar, IMR90 cells [21]; blue bars, MCF7 cells [20]. The external data are derived from single ChIP-seq experiments, thus ‘Total’ peaks were used. **(E)** Proportion of CGI-type peaks vs. all peaks within the promoter regions. **(F)** Canonical p53 motif enrichment determined by p53 specific position weight matrices. **(G)** Histogram of p53 motif occurrence position distribution in HC-peak as determined by PscanChip in the RIS condition. Frequency of p53 motifs in each 50 bp window across 6148 CpG and 3700 non-CpG peaks is plotted across +/- 1000 bp regions around the TSS. **(H)** Genome browser snapshot of ChIP-seq for p53 and the indicated histone marks at the *p21* locus in each condition. The vertical lines labeled #1 and #2 indicate the classical distal and proximal p53REs. Two representative RefSeq transcripts encode the same protein. The vertical scaling of ChIP-seq tracks for each antibody is identical between conditions.

We next examined whether this phenotype-associated gene regulation was achieved through a specific p53 binding profile by using p53 ChIP-seq analyses of the acDDR, RIS and pApo conditions compared with normal-growing cells (S2 Table). We used at least three replicates for each condition (except the growing condition, with two replicates) to define high-confidence (HC) peak sets (see materials and methods). In contrast to the strong induction of p53 during acDDR, actual peak numbers were substantially lower than in the other conditions (Fig. 2A). The number of HC peaks in the acDDR condition was comparable to peak sets described in earlier reports [12,13,19–21]. Notably, as in our acDDR condition, these studies were performed on cells treated for less than 24h. Our data suggest that the mode of p53 exposure, acute or chronic, affects the affinity of p53 binding and therefore the outcome. The genomic features of the HC p53 binding sites in the acDDR condition differed from those in RIS and pApo. The proportion of p53 peaks that mapped to transcription start site (TSS) proximal regions (core promoter) was substantially higher in the RIS and pApo conditions at 64% and 50%, respectively, compared with only 26% in the acDDR condition, where the majority of peaks (>70%) were in introns, exons or up-stream distal regions (Fig. 2B). The preferential association of p53 with promoter regions in the chronic conditions is not due to varied numbers of p53 peaks between conditions, because the association was conserved when we selected the same numbers of peaks from each condition for the analysis (S3 Table).

Through visual inspection of our ChIP-seq data using a genome browser, we noticed that p53 peaks tended to be either sharp or broad, with acDDR peaks being substantially narrower than in the other conditions (Fig. 2C). There are two major types of core promoter: ‘focused’ with a single or a few densely aggregated TSSs, and ‘dispersed’ with many TSSs. In vertebrates, these ‘focused’ and ‘dispersed’ promoters typically correspond to non-CGI-promoters containing core promoter elements (e.g. TATA-boxes) and CGI-type promoters, which are generally TATA-less, respectively [22]. We examined the co-occurrence of p53 peaks and CGIs in each condition. p53 peaks in the chronic (RIS and pApo) conditions overlapped substantially more with CGIs than in the acDDR condition. The acDDR-associated peaks in our and other published p53 datasets were mostly of the non-CGI type (Fig. 2D). The higher frequency of CGI-type p53 peaks in chronic conditions is not simply due to their preferential distribution in the promoter regions (Fig. 2B), since this tendency was retained when examining promoter regions only (Fig. 2E). These data show the distinct genomic binding profiles of p53 between the acute and chronic conditions, revealing extensive usage of CGI promoters in the latter.

Gene ontology (GO) enrichment of non-CGI p53 peaks mapped to genes showed that the functional groups involved in the typical p53-associated functions, such as cell cycle, DNA damage and apoptosis, were overrepresented particularly in the chronic conditions, whereas for the CGI p53 peaks, we observed the most significant enrichment for functional groups involved in RNA metabolism and processing (S4 Table). These data suggest that the outcome of p53 binding in the chronic conditions is different from that of the acute condition, which has been a commonly used experimental system, and thus our data substantially extend not only the list of candidate p53-targets and but also their mode of regulation.

We next examined whether these p53-bound regions contained the p53 consensus motif. Using position weight matrices, searching for known canonical p53 responsive elements (p53REs), we identified their enrichment in both types of peaks (Fig. 2F, see Materials and Methods). Reflecting the peak shapes, p53-binding motifs were dispersed throughout the CGI-type peaks, whereas p53REs were focused around the peak center of non-CGI-type peaks (Fig. 2G).

Such CGI-type p53 peaks have not been reported even in the promoter of *CDKN1A* (*p21*), the best-characterized p53 target (Fig. 2H). The well-established view is that *p21* has two major canonical p53REs at around-2.3 kb (the distal p53RE) and-1.4 kb (the proximal p53RE). The distal p53RE is bound more strongly by p53 than the proximal site [23]. We consistently observed sharp p53 peaks at the distal site in all conditions (#1 in Fig. 2H). In addition, the *p21* locus contained prominent p53 enrichment at the major CGI, which encompasses the classic *p21* TSS, in chronic conditions only. p53 binding to the CGI, which contained various potential p53REs (Figs. 2 and S2A), coincided with enrichment for H3K4me3 (a marker of CGI-promoters) and a downstream spreading of H3K36me3 (a marker for transcription elongation) [24] (Fig. 2H), suggesting that this CGI is a promoter for the classic *p21* transcript variant 1 (v1). Both the classic v1 and the alternative transcripts—represented by variant 2, whose TSSs are located in direct proximity to the distal p53RE (#2 in Fig. 2H)—were up-regulated in all conditions, therefore the relative contribution of the distal p53RE and the CGI promoter to *p21* v1 is not yet clear (Figs. 2 and S2B-S2C). Nevertheless these data reinforce the unexpected association between chronic p53 and CGI promoters.

### Chronic p53 comprises extensive self-regulatory hubs

We next compared our p53-dependent expression data with our p53 binding data. In contrast to the expression profiles (Fig. 1F), the overlap in the p53 binding profiles between conditions was substantially larger, and the similarity was even more striking for the peaks within the promoter regions (S3A Fig). To better predict phenotype-associated p53 function, we developed the “R-based analysis of ChIP-seq And Differential Expression” (Rcade) package, integrating genome-wide binding profiles of TFs with their responsive gene expression profiles. Briefly, we coupled the expression analysis to a TSS-local read-based ChIP-seq analysis, thereby circumventing ‘peak-calling’ and thus reducing false-positives and bias issues inherent with peak-calling methods. However, because most of the acDDR peaks failed to fulfill the localization criteria specified (S3B Fig), in our further analyses we only focused on the pApo and RIS chronic conditions, where Rcade identified 1487 and 563 genes, respectively, which included both established and many previously unknown, ‘putative’ p53 targets (S3C Fig and S5 Table). GO analysis of the Rcade-derived genes showed that various biological processes were represented in both conditions, including typical p53-related functions (cell cycle, DNA damage response, and apoptosis); functions of membrane-bound organelles and metabolism; and gene expression and RNA metabolism/processing (S3D Fig).

The Rcade-derived genes include both previously known as well as many unknown/uncharacterized genes as direct targets of p53. For example, *ANKRA2* and *HSPA4L*, which are poorly characterized, were identified as putative direct p53-inducible targets in both RIS and pApo conditions. Significant down-regulation of *ANKRA2* and *HSPA4L* upon p53 knockdown was confirmed by qPCR in at least two different conditions in IMR90 cells (S3E Fig). Similar results were obtained using the second sh-p53 (S3E Fig). Interestingly, tumor-specific, disruptive mutations of *ANKRA2* were previously identified in oral squamous cell carcinoma [25], and mutations in *ANKRA2* are also reported in the Catalogue Of Somatic Mutations In Cancer (COSMIC, http://www.sanger.ac.uk/genetics/CGP/cosmic/). In addition, methylation of the CGI promoter of *HSPA4L* as well as the methylation-associated down-regulation of *HSPA4L* in acute lymphocytic leukemia (ALL) have been reported previously [26], thus underlining the usefulness of our Rcade datasets. Using a *PiggyBac* transposon system [27], we established a tetracycline-inducible p53 system in H1299 cells (a p53-null lung cancer cell line) and confirmed that ectopic wild type p53 could induce expression of *ANKRA2* and *HSPA4L* (S3F Fig).

**Fig 3:**
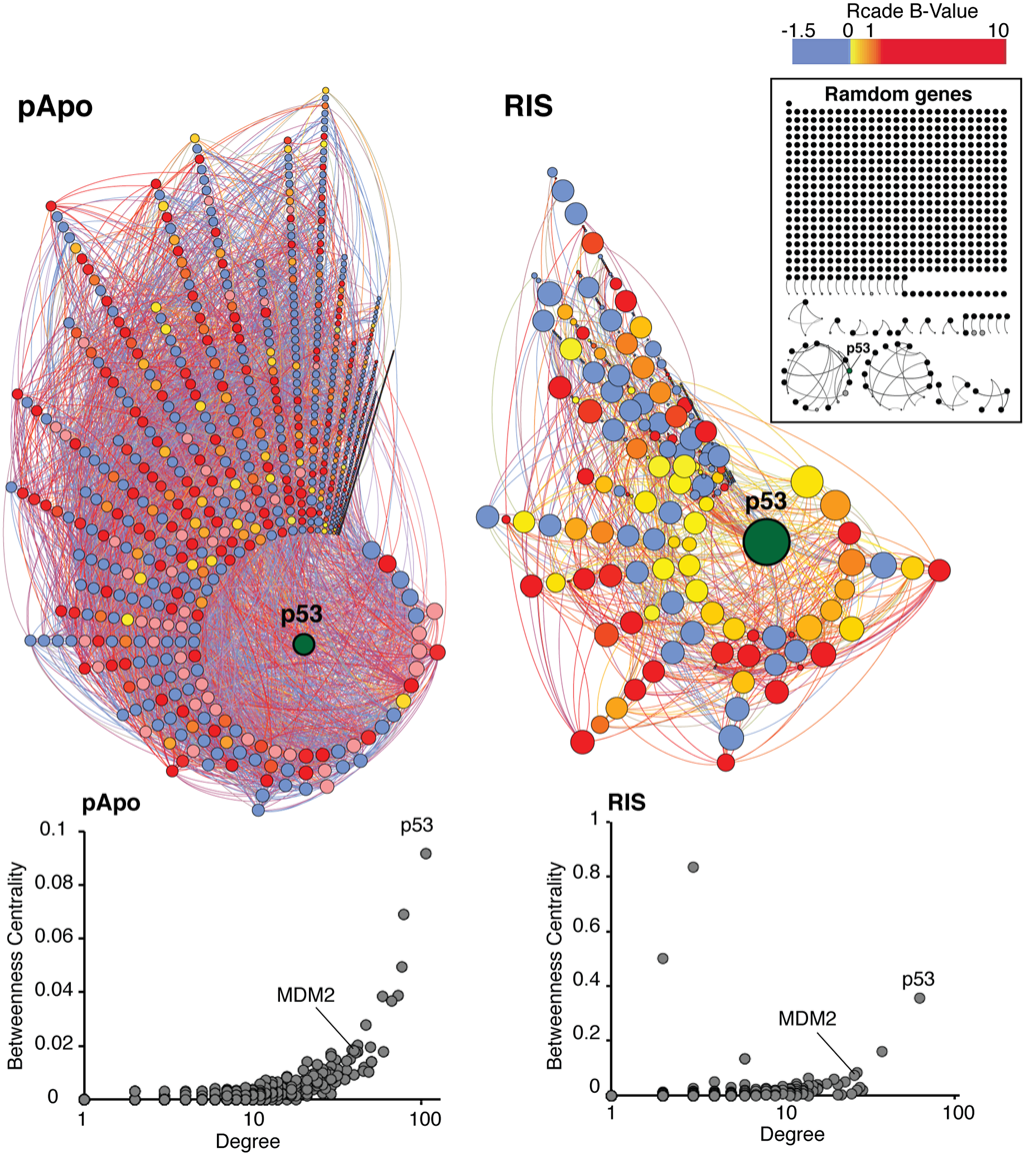
High connectivity within the (putative) p53 direct target genes. Integrative networks of Rcade-derived p53 targets were generated utilizing >300 external high-throughput genomic and proteomic datasets. Nodes are colored by the Rcade B-value, which represents the probability of genes being direct p53-targets. Nodes are spatially organized by ‘Degree’ (local connectivity) and the size of the nodes represents their ‘Betweeness centrality’ (global connectivity). Edge colors indicate the data types used. p53 represents the central node (the highest Degree) in both conditions. A random list of genes (including p53) was chosen to generate a control network using the same methods (inset). For each condition, a graph plotting the two network topology measures (global and local) was generated, representing overall connectivity. For simplicity only Rcade genes positively regulated by p53 are shown.

To gain a comprehensive understanding of the p53 regulome, we first generated integrative networks of the Rcade-derived p53-targets, taking advantage of numerous external high-throughput datasets. Since co-regulated genes are likely to be ‘connected’, we measured connectivity within the Rcade-derived p53-targets, taking into account topological measures of local (‘Degree’) and global (‘Between-ness centrality’) connectivity (see materials and methods). This largely unbiased network approach revealed that the (putative) p53-targets were highly inter-connected, providing evidence for the validity of our Rcade gene lists (Fig. 3, compare to the random gene set). p53 was identified as the most globally (pApo) and locally (both conditions) connected gene in the networks, indicating the importance of p53 to the integrity of entire networks.

To gain insight into the functional relationship between putative p53 targets, we next constructed phenotype-specific, ‘knowledge-based’ pathway models of the p53 regulome (see materials and methods) (S4 and S5 Figs, high resolution figures are available at http://australian-systemsbiology.org/tp53). These revealed a highly complex network in the pro-apoptotic condition and provided the first detailed p53 regulome of senescence. p53 appeared to regulate multiple components within the same pathways or biochemical complexes, but often with distinct aspects depending on the cellular context. Thus many p53-related phenomena fragmented throughout the literature could be seen in a single biological context, and yet each context may involve distinct p53 functions. For example, Rcade genes associated with mitochondria in the pApo condition were largely distinct from those in the RIS condition and included, in addition to apoptotic genes, genes involved in mitochondrial metabolism and homeostasis (oxidative phosphorylation, fatty acid and lipid metabolism, mitochondrial biogenesis). Consistent with a recent study, which showed an extensive transcriptional regulation of autophagy by p53 in response to acute DNA damage in mouse embryonic fibroblasts [13], we also found that the autophagy program was regulated by p53 in the chronic conditions (pApo in particular) but through largely distinct genes compared to the previous report [13] (S4 Fig), extending the role for p53 in autophagy regulation.

**Fig 4:**
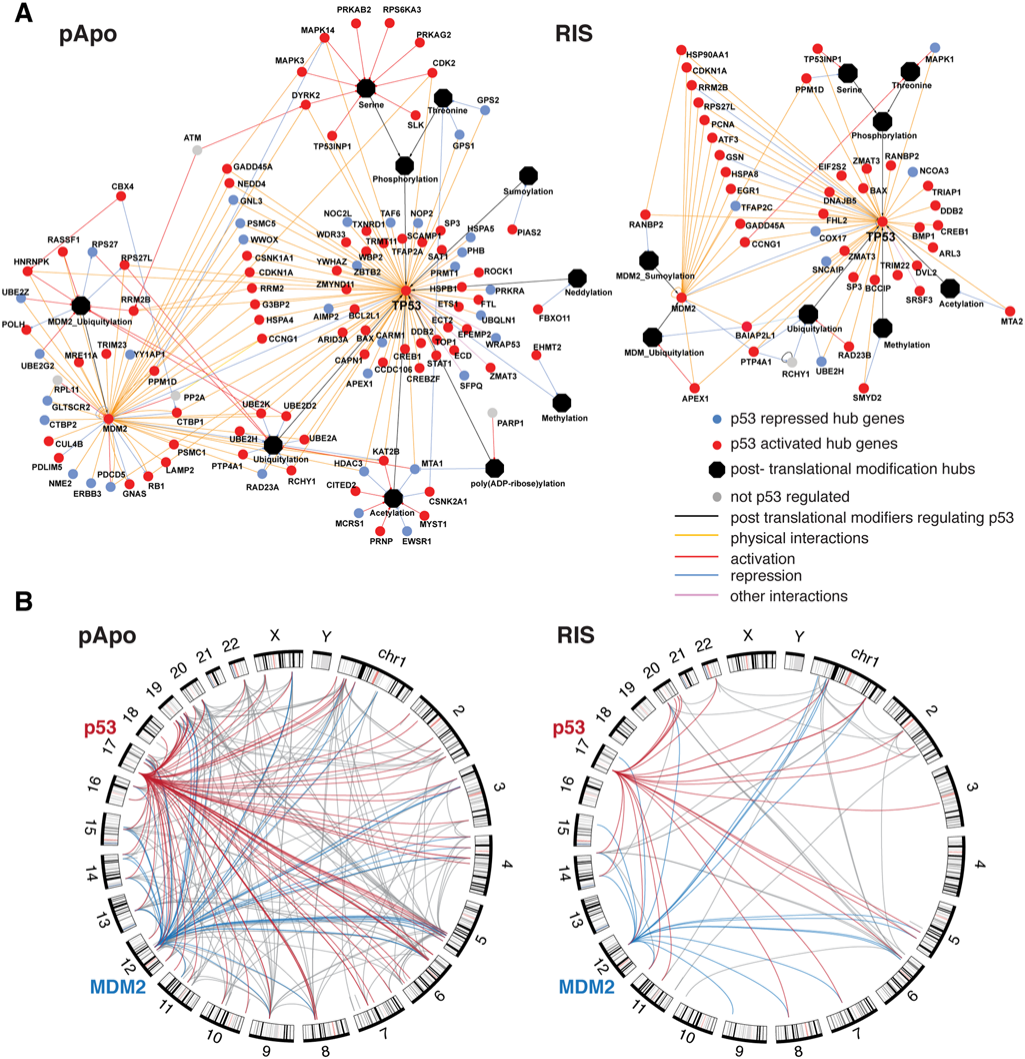
The p53 regulome reveals an extensive self-regulatory hub. **(A)** Hubs were generated by integrating (putative) p53 targets from Rcade analysis with pathway information, protein-protein interactions and literature mining. **(B)** Circos plots showing physical interactions between all hub genes. Red, p53-interactions; blue, MDM2-interactions; gray, other component-interactions.

One striking notion from our pathway modeling is that a subset of the p53 regulome formed a ‘p53 hub’: p53 has been reported to interact with, or be modified by, the components of this hub in diverse experimental conditions, thus suggesting that many of the direct targets of p53 in turn regulate p53 in the chronic conditions (Figs. 4A, S4, and S5, and S6 Table). This is in accordance with the high local connectivity of p53 in the networks. Information specifically about protein-protein interactions between the p53 hub components highlighted that many of them can interact with each other (Fig. 4B). The components within the self-regulatory network of p53 are best exemplified by MDM2, the E3 ubiquitin ligase, which negatively regulates p53 stability, thereby conferring a strong negative feedback loop [7]. However, an MDM2-independent negative feedback loop has been shown in a senescence context [28]. Moreover, additional mechanisms for modulating the MDM2-p53 loop are suspected to exist in the cancer context [29,30]. Of note, consistent with the high connectivity of MDM2 in our p53 networks (Fig. 3), MDM2 itself formed a prominent ‘sub-hub’ within the p53 hub (Fig. 4A), reinforcing the existence of multiple levels of mechanisms for regulating p53 and the p53-MDM2 loop in the chronic conditions. Together, our data suggest that intensive and multi-level fine-tuning of p53 function may be an important mode of phenotype regulation.

### p53 represses the lipogenic enzyme SCD in RIS, pApo, and cancer

**Fig 5:**
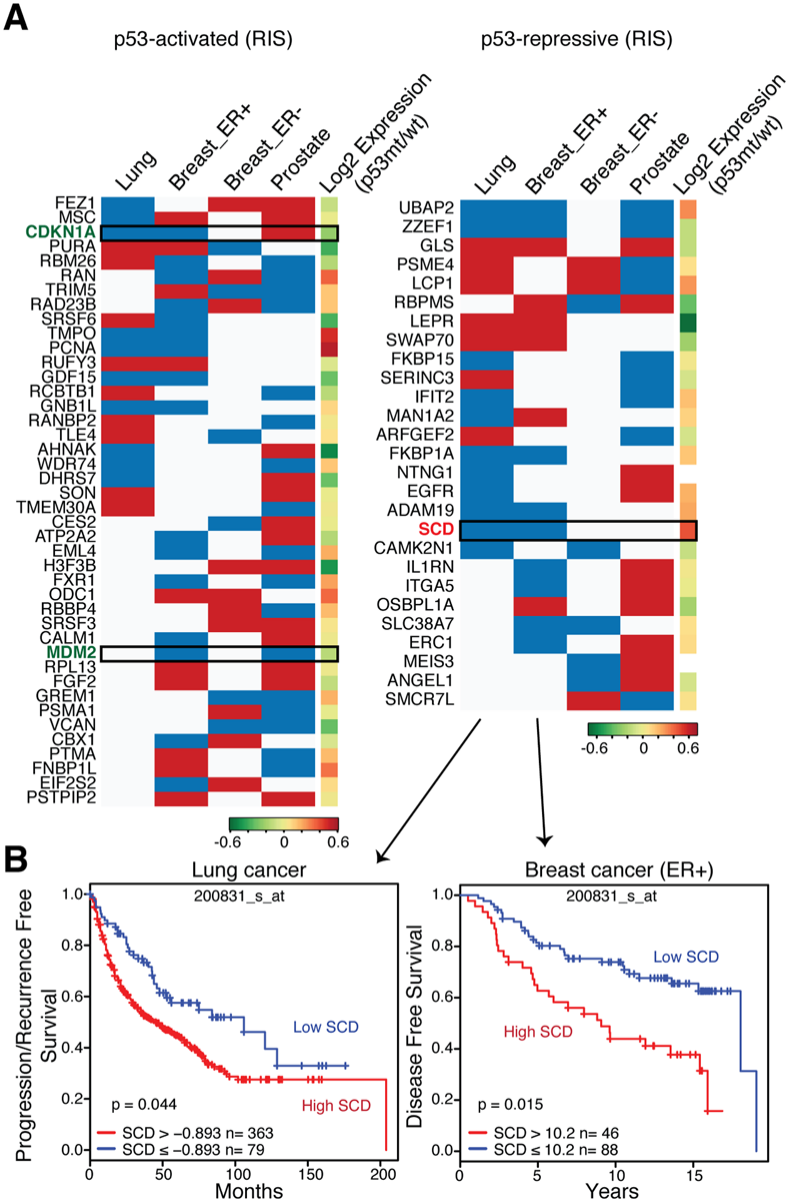
Prognostic role of Rcade-derived p53-targets in cancer. **(A)** Heatmap summarizing RPA for Rcade-derived p53 targets in the RIS condition for four cancer datasets indicated: Lung [31], Breast_ER+ and ER- [32], Prostate [33]. Blue and red denote worse and better survival, when expression of genes is high. The fifth column depicts the ratios of gene expression in p53 mutant (mt) vs. wild-type (wt) tumors in separate breast cancer datasets [38]. *CDKN1A* (*p21*), *MDM2*, and *SCD* are highlighted. *SCD* was also p53-repressive in the pApo condition. **(B)** Kaplan–Meier plots in the indicated cohorts for patients with high and low *SCD* levels.

Finally, to test the clinical relevance of our datasets for chronic p53 targets, we performed recursive partitioning analysis (RPA) of each Rcade component for survival in four publicly available cancer datasets (Fig. 5A) [31–33]. For example, the RPA identified an association between high levels of *MDM2*, a *bona fide* oncogene, and poor prognosis in two datasets (Fig. 5A). On the other hand, we observed a mixed association between prognosis and *p21* (*CDKN1A*) levels, whose clinical relevance in human tumors is controversial, supporting the validity of this method [34] (Figs. 5A and S6). Interestingly, several autophagy genes were identified in the pApo condition, where high levels of these genes were mostly associated with better prognosis in multiple clinical datasets (S6 Fig, left). Implications of autophagy in cancer are complex and thus careful interpretation is necessary, but these data support the recent study that showed the contribution of autophagy to p53-dependent tumor suppression [13]. Using this method we went on to validate clinically relevant p53 putative targets. We prioritized p53-repressive targets, since p53 mutations are common in cancers where p53-repressed genes are likely to be up-regulated, and if those gene products contribute to tumorigenesis, they may provide good candidates for therapeutic targets in p53-deficient cancers. Of the p53-repressive targets whose expression levels were significantly correlated with prognosis in at least two different datasets, we chose the lipogenic enzyme stearoyl-CoA desaturase (SCD) for further validation, for the following reasons (Fig. 5A): ‘lipid metabolism’ was featured in our pathway modeling in both chronic conditions (S4 and S5 Figs.); the ‘lipogenic phenotype’ is a hallmark of cancer [35]; high *SCD* expression has been correlated with a transformation phenotype, tumor cell survival, and poor outcome in many cancers, and SCD has been implicated as potential targets for cancer therapy [36]. Although several lipogenic TFs, such as SREBFs and PPARs, have been implicated in the regulation of *SCD* expression, it is not clear how SCD is regulated under stress as well as in cancer [37].

**Fig 6:**
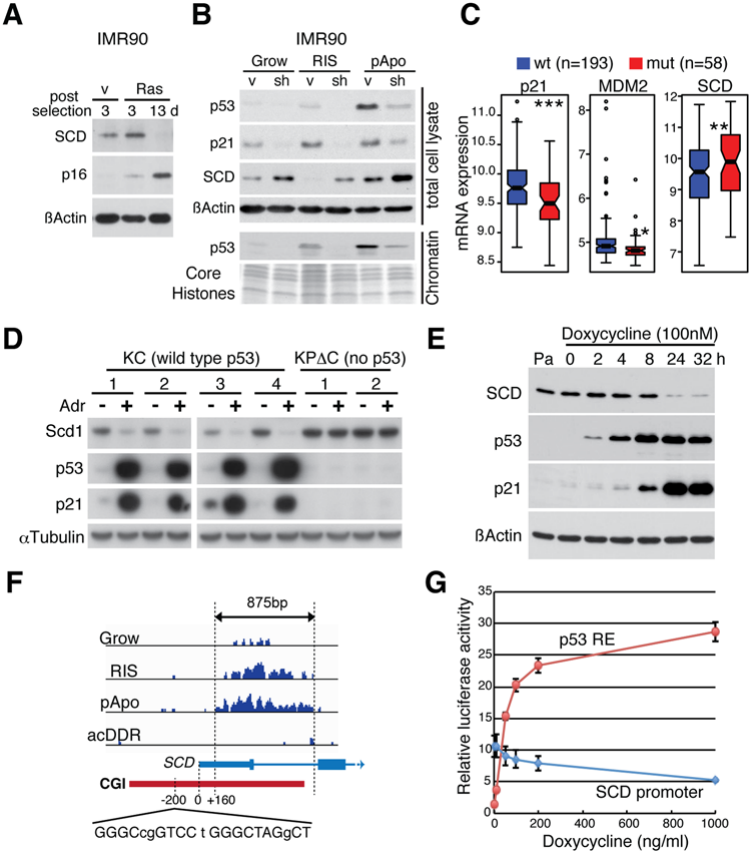
The lipogenic enzyme SCD is a direct p53-repressed target. **(A)** Immunoblot analysis showing dynamic regulation of the SCD level during RIS in IMR90 cells. SCD was initially up-regulated at as early as day 3 post selection for oncogenic RAS expression but eventually down-regulated when senescence was fully established (see p16 levels). **(B)** Immunoblot analysis for indicated proteins in indicated conditions. SCD was up-regulated upon p53 knockdown (sh-p53#1) in both RIS and pApo conditions. The assay was performed at d10 post-puromycin selection for RAS. **(C)** Quantitative representation of the correlation between expression of the indicated genes and p53 status in the dataset [38] used in (A). *p < 0.05, **p < 0.01, ***p < 0.001. **(D)** Immunoblot analysis for indicated proteins in Kras-driven mPDA cell lines. Four cell lines (KC1–4) are p53 wild-type and two cell lines (KPΔC1–2) are p53-null background (see Materials and Methods). Cells were treated with 0.5 μg/ml Adriamycin (Adr) for 12h. **(E)** Immunoblot analysis for the proteins indicated, in p53-inducible H1299 cells at the indicated time points after doxycycline addition. Pa, parent H1299 cells. **(F)** Genome browser snapshot of p53 ChIP-seq at the *SCD* locus. A part of the *SCD* RefSeq transcript with the arrow indicating the direction of transcription. Red bar, CGI. It was previously shown that the upstream canonical p53RE at-200bp from the TSS can be bound by overexpressed p53 by ChIP-qPCR [40], but we found no substantial association of endogenous p53 with this p53RE (mismatches in the decamers and the spacer between two decamers are shown in lowercase). The vertical scaling is identical between conditions. **(G)** Relative luciferase activity in p53-inducible H1299 cells co-transfected with a luciferase reporter containing the *SCD* promoter mapped by our p53 ChIP-seq (the region between the dotted vertical lines in (F)) or a synthetic p53RE with the constitutive Renilla luciferase-expressing plasmid.

SCD catalyzes the rate-limiting reaction in the biosynthesis of the major monounsaturated fatty acids (oleate and palmitoleate), which are components of essential building blocks of rapidly proliferating cells [37]. Consistently, SCD was initially up-regulated in response to hyperactive RAS, and then it reduced to an almost undetectable level after the full establishment of senescence, where p16, a marker of senescence, is highly up-regulated (Fig. 6A). In E1A/RAS expressing transformed pApo cells, SCD levels were relatively high, supporting the role of SCD in rapidly proliferating transformed cells (Fig. 6B). In both cases, however, when we introduced sh-p53 to RIS or pApo cells, SCD levels were up-regulated, suggesting that SCD is regulated by multiple mechanisms, whereby p53 counteracts the positive control of SCD by pro-tumorigenic signals.

To lend further support to the finding that *SCD* is repressed by p53 in cancer, we analyzed a publicly available breast cancer dataset that contains gene expression and p53 sequencing data [38]. In contrast to *p21* and *MDM2*, *SCD* levels were significantly higher in tumors with p53 mutations than with wild-type p53 (Fig. 6C). We also examined the relationship between p53 and Scd1 (a mouse homologue of SCD) in Kras-driven mouse pancreatic ductal adenocarcinoma (mPDA) cell lines established from *Kras* ^*LSL-G12D*^; *Pdx1-cre*, or *Kras* ^*LSL-G12D*^; *P48-cre mice* (KC cell lines) and *Kras* ^*LSL-G12D*^; *p53lox/+*; *Pdx1-cre* compound mutant mice (KP^Δ^C cell lines) [39]. In KC cell lines (p53-wild type), p53 was readily up-regulated by DNA damage treatment, whereas p53 was undetectable in KP^Δ^C cell lines (p53-null) (Fig. 6D). Scd1 was down-regulated in the KC, but not in the KP^Δ^C, cell lines (Fig. 6D). Furthermore, repression of SCD by p53 was confirmed in a tetracycline-inducible p53 system in H1299 cells. Upon doxycycline addition, the endogenous SCD level was repressed in a dose- and time-dependent manner (Figs. 6E and S7A). In the *SCD* locus, chronic p53 accumulation was observed mainly on the CGI (Fig. 6F). Although an early study showed that overexpressed wild type p53 can bind the upstream canonical p53RE in the *SCD* promoter (Fig. 6F) [40], our data indicate that endogenous p53 preferentially accumulates on a distinct region in the *CGI* promoter when it is chronically activated. This p53-bound region, containing several p53 motifs (Figs. 6 and S7B), was sufficient for p53 to repress downstream luciferase expression (Fig. 6G). Taken together, our data suggest that *SCD* expression, which is associated with poor prognosis in some cancers, is directly repressed by chronic p53 through the CGI promoter, providing direct mechanistic insight into the anti-lipogenic role of p53.

## Discussion

Here we present an extensive study of p53 regulation in different phenotypes using normal human cells. We compared p53 binding profiles in three settings; acDDR, RIS, and E1A and RAS-expressing pApo conditions. In the acDDR condition, which has been the commonly used model for genome-wide mapping of p53 binding sites, p53 peaks were primarily of a sharp non-CGI type, exhibiting a wide distribution in the genome. Interestingly, increasing evidence for distant gene regulation by p53 has been shown using systems where p53 is acutely activated [14,41]. This may explain, in part, the diverse locations of non-CGI p53 peaks in the acDDR condition. In contrast, both RIS and pApo conditions were associated with sustained accumulation of p53 on chromatin, where p53 preferentially associated with CGI promoters. In one of the previous p53 ChIP-seq studies, Botcheva et al. identified a substantial number of CGI-type p53 peaks in an acute condition (Fig. 2D) [21]. We reanalyzed these external data and found 1811 p53 CGI-peaks, 50% and 52% of which were included in our HC p53 CGI-peaks in the RIS (6148 CGI-peaks) and pApo (6566 CGI-peaks) conditions, respectively. Although the relatively high frequency of CGI-peaks in this external dataset (compared to 846 HC p53 CGI-peaks in our acDDR condition) may be an overestimate due to their lack of biological replicates, it reinforces the significance of the connection between p53 and CGI promoters. It is not clear why their study identified many CGI peaks in their acDDR condition. Both studies used HDFs (IMR90 cells), which are highly sensitive to senescence induction by oxidative stress. Notably, we maintained our cells in a physiological (5%) O_2_ condition to minimize the amount of oxidative stress derived from routine cell culture. Thus the basal levels of p53 and the background senescence phenotype might be different between the studies.

The molecular mechanism for the unique profile of chronic p53 seen in our study is unclear. The levels of global chromatin bound p53 were comparable between the acute and chronic (at least RIS) conditions (Fig. 1E). Furthermore, p53 binding profiles at promoter regions were almost identical between the RIS and pApo conditions, but the Rcade gene sets were distinct (compare S3A and S3C Figs). Thus, quantitative differences in the global levels of p53 or its genomic distribution alone cannot explain the differential p53 activities.

Generally, CGIs are ‘open’, enriched for the binding sites of many TFs, including Sp1, which can recruit the TATA-binding general TF complex to TATA-less CGI promoters [22]. Thus in CGI regions, it is conceivable that complex interactions between transcription (co)factors can occur depending on cellular contexts. The consensus p53 binding site consists of two decameric half-sites separated by 0–13 nucleotides, but the ‘non-canonical’ half-sites can also function as a p53RE [42,43]. Our analysis of two CGI promoters, which are p53-activated (*p21*) and p53-repressive (*SCD*), suggests that both CGI-promoters contain multiple ‘weak’ p53REs (including many half-sites), which somehow favor persistent accumulation of p53 (S2A and S7B Figs). These weak p53 associations might well be reinforced by other factors. It is also possible that p53 might associate with DNA through its binding partners. Indeed, our motif enrichment analyses identified known p53-cofactors, including Sp1 (S7 Table) within p53 CGI-peaks. Therefore, it is possible that persistent cellular stress creates distinct contexts, where the quality of p53 (e.g. its post-translational modifications, PTMs) and the sets of p53 binding proteins are different from acute conditions, thereby facilitating the p53-CGI association. Indeed, p53 can be modified by a multitude of diverse PTMs, including phosphorylation, acetylation, methylation, ubiquitilation, neddylation, sumolyation, and poly-ribosylation [44]. Although the functional roles of these PTMs are not fully understood, some PTMs such as phosphorylation and acetylation typically contribute to stabilization and activation of p53 [44]. Interestingly, as shown in Fig. 4A, many factors involved in PTMs of p53 were included in the p53 self-regulatory hubs derived from the Rcade gene sets (Fig. 4A). This might provide a mechanism for context-dependent fine-tuning of PTMs of p53 at least at a global level. It will be important to determine phenotype-specific genome-wide profiling of individual PTMs of p53. In addition, a recent study has shown that a genome-wide redistribution of DNA methylation occurs during replicative senescence, where persistent p53 plays a key role [45]. Thus it would also be interesting to examine the structural alterations in CGI regions during RIS and pApo conditions.

Notably, these two chronic phenotypes are highly distinct; RIS cells are stably arrested and resistant to apoptosis, whereas pApo cells are rapidly proliferating and sensitive to apoptosis, yet both are largely dependent on p53 [16,17]. Such distinct p53-associated phenotypes were not achieved through differential p53 binding alone, since both conditions exhibited highly similar p53-binding profiles, where CGI-type genes are over-represented (S3A and S3C Fig). The unique feature of CGIs, such as their relatively open configuration and their enriched TF binding motifs, might also provide environments that allow for diverse downstream regulation upon p53 binding in conjunction with other (co)factors [46]. In addition, our integrated network analyses in chronic conditions identified the extensive capability of p53 for physical interaction with its own targets, further reinforcing the diverse results of p53 binding to the same target promoters. Although the dynamic regulation of p53 through the MDM2-p53 negative feedback loop was readily detected in the DDR condition (Fig. 1C), its relevance in the chronic conditions was not so obvious. In pApo transformed cells, MDM2 was highly up-regulated compared to other conditions, whereas the chromatin bound p53 levels were comparable, or even slightly higher in the pApo condition (Fig. 1D). Although this may be in part due to E1A-induced p14ARF, which inhibits the E3 ligase activity of MDM2 [47], this is also reminiscent of the tumor specific escape of mutant p53 from Mdm2 degradation in mice harboring germ line p53 mutations, an observation that suggests the existence of additional mechanisms for modulating the p53-MDM2 loop during tumorigenesis [29,30]. It has also been shown that the p53-repressive target, malic enzyme 2, reciprocally suppresses p53 in an MDM2-independent manner during senescence [28]. Together, the dysregulation of p53-hubs particularly in chronic conditions might be a critical step for tumorigenesis.

**Fig 7:**
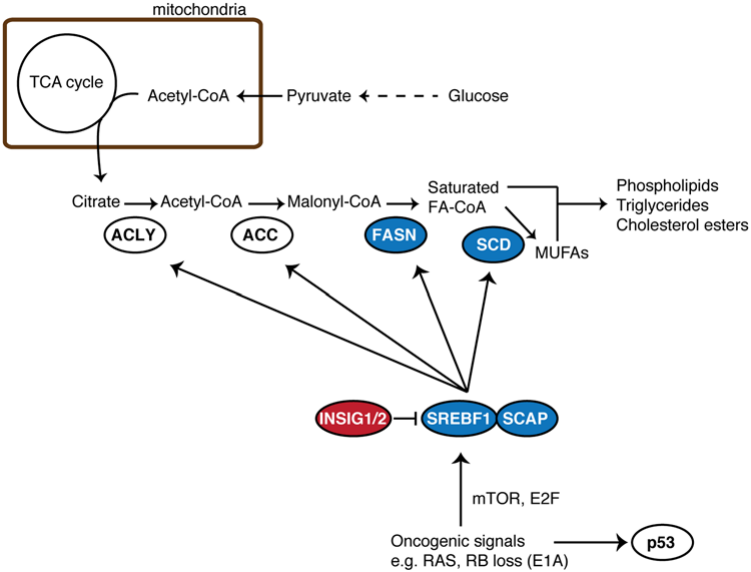
Simplified schematic of *de novo* fatty acid synthesis. Gene products of Rcade-derived p53-targets are colored. Red and blue represent genes positively and negatively regulated by p53, respectively. Oncogenic signals activate fatty acid (FA) biogenesis in part through the activation of SREBF1. Typically oncogenic signals also stimulate the p53 pathway. In addition to *SCD*, we found several Rcade genes (at least in one condition) involved in this process, suggesting that p53 regulates *de novo* FA synthesis at multiple levels. FA-CoA, fatty acyl-CoA; MUFAs, monounsaturated fatty acids.

The complex and multi-level gene regulation by chronic p53 appears to apply to its regulation of genes involved in fatty acid synthesis. SCD was previously shown to positively regulate p53 transcription [48], thus SCD may be a part of the self-regulatory p53 hub. In addition to *SCD*, Rcade genes included many other genes involved in lipid metabolism in at least in one condition, indicating that p53 regulates fatty acid metabolism at multiple steps (Fig. 7). Consistently, recent metabolomics studies showed that senescence can be associated with reduced lipid synthesis and increased fatty acid oxidation [49,50]. The Rcade genes associated with lipid metabolism include *FASN* and *SREBF1* (also known as *SREBP1*), which were repressed by p53. *FASN*, which encodes another key lipogenic enzyme, is typically co-regulated with *SCD* by the lipogenic TF, SREBF1, and *FASN* was previously shown to be a target of the p53 family members, p63 and p73 [51]. It was also shown that ectopic p53 can repress the promoter activity of *SREBF1* [52]. We confirmed their repression by ectopically expressing p53 in H1299 cells, suggesting that, together with our Rcade analyses, *FASN* and *SREBF1* are also direct p53-repressive targets (S7C Fig). Importantly, the levels of SREBF1 were not reduced but rather slightly up-regulated in the chronic conditions (S7D Fig), thus it is likely that repression of SCD expression by p53 in these settings is direct (Fig. 6B). Given the dynamic regulation of SCD during RIS and pApo, sustained p53 might compete with SREBF1 (or other lipogenic factors) at CGI regions. Interestingly, a recent study showed that mutant p53 positively regulates lipogenic genes, including *SCD* and *FASN*, in an SREBF1-dependent manner [53]. This study reinforces not only the anti-lipogenic role of p53 but also the functional link between p53 and SREBF1 in lipogenic gene regulation. In addition, it has been shown that p53 is up-regulated in the adipocytes of obese mice, where p53 negatively regulates SREBF1 [52]. It is possible that chronically activated p53 acts as a counter measure against excessive and tumorigenic fatty acid synthesis through various mechanisms. Altogether these results provide additional mechanistic insight into p53 tumor suppression, suggesting that our data represent unique tools for finding cancer therapeutic targets in a p53-mutant context.

## Materials and Methods

### Cell culture and vectors

IMR90 cells (normal human diploid fibroblasts) (ATTC) were cultured as previously described under the 5% O_2_ condition [54]. H1299 cells (p53-null lung cancer cells) (ATCC) and mouse pancreatic ductal adenocarcinoma (PDA) cell lines were cultured in DMEM with 10% fetal bovine serum (FBS) under ambient oxygen levels. The PDA cell lines KC1 (T4878), KC2 (TB1572) and KC3 (T9394) were established from Kras^LSL-G12D^; Pdx1-cre (T4878 and T9394), and Kras^LSL-G12D^; P48-cre (TB1572) mice as described previously [39]; KP^Δ^C was established from Kras^LSL-G12D^; p53^lox/+^; Pdx1-cre compound mutant mice, generated after breeding with Kras^LSL-G12D^ [55], Pdx1-cre [56] and p53^lox^ [57] strains.

The following retroviral vectors were used in this study: pBabe-Puro (HRAS^G12V^), pWZL-Hygro (E1A, HRAS^G12V^), and pLNCX2-Neo (ER:HRAS^G12V^, encoding a fusion protein of the estrogen receptor ligand-binding domain and H-RASV12) [54]. The lentiviral RNAi, using a miR30 design, has been described previously [18]. Target sequences of sh-p53: GAGGATTTCATCTCTTGTA (sh-p53#1) [18] and CACTACAACTACATGTGTA (sh-p53#2). To examine p53-dependent gene expression in each condition, sh-p53 was introduced after the establishment of the phenotype and samples were collected after 5 days, except for the acDDR condition, where sh-p53 was introduced first for at least 5 days before the administration of etoposide (100 μM for 24h).

The tetracycline inducible system (pCLIIP-i) for p53 was built into a PiggyBac transposon system [27] in two stages. The first stage plasmid comprised the minimal transposon pCyl50 (provided by the Wellcome Trust Sanger Institute, Hinxton, United Kingdom) [58] with a linker, HS4 transcriptional insulators and a PGK-puro expression cassette. The tet-inducible components were added, with a third generation tet-responsive element [59] and a constitutively expressed rtTA3 tet-transactivator (derived from pTRIPZ; Open Biosystems). Wild-type human p53 cDNA was cloned downstream of the tet-responsive element (pCLIIP-i-p53).

### Establishment of p53-inducible H1299 cells

p53-null H1299 human lung cancer were co-transfected with pCLIIP-i-p53 with the mouse codon-biased *PiggyBac* transposon (*mPB*) gene. H1299 cells stably expressing pCLIIP-i-p53 were established in puromycin (1.5 μg/ml) containing media for 7 days, and then maintained in the puromycin-free medium.

### Luciferase assay

Luciferase activity was assayed using Dual-Luciferase Reporter Assay System (Promega) according to the manufacturer’s instructions. Reporter plasmids were transfected to p53-inducible H1299 cells. After 48h of transfection, doxycycline was added to induce p53 expression. Cells were lysed in passive lysis buffer after 24 hours of doxycycline treatment and luciferase activities were measured using a PHERAstar FS microplate reader (BMG LABTECH). The p53-enriched region in the *SCD* locus in the RIS condition (Figs. 6F and S7B) was synthesized (GeneArt), and cloned into the pGL4.15 luciferase reporter plasmid (Promega) between KpnI and XhoI sites. pGL4.38 (Promega), which contains 2x tandem synthetic p53RE, was used as a positive control. The thymidine kinase promoter-Renilla *luciferase* reporter plasmid (pRL*-*TK) was used as a normalization control.

### Chromatin isolation

Chromatin isolation was performed as described before [18].

### Immunoblotting

The following antibodies were used for immunoblotting: anti-HRAS (Santa Cruz, sc-29), anti-human p21 (Santa Cruz, sc-397), anti-E1A (Santa Cruz, sc-430); anti-ß-actin (Sigma A5441), anti-Cyclin A2 (Sigma C4710), anti-human p53 (DO-1, Sigma P6874), anti-MDM2 (clones 2A10 and 4B11) [18], anti-Histone H3 (Abcam ab1791), anti-HMGA2 (Santa Cruz, sc-30223), anti-SCD/Scd1 (Cell Signaling, #2438), anti-mouse p53 (Biovision #3036) and anti-mouse p21 (Santa Cruz #sc-6246), anti-α-Tubulin (Abcam #Ab18251), anti-SREBP1 (Santa Cruz, sc-13551). Immunoblotting analysis was carried out as described [18].

### Cell proliferation, SA-ß-Galactosidase (SA-ß-Gal) and cell viability assays

Replicating DNA was labeled using BrdU, and SA-ß-Gal activity was assessed as described [18]. Cell viability was determined using a trypan blue exclusion assay.

### Quantitative RT-PCR

RT-qPCR was performed as described before [18]. *CDKN1A* (*p21*) variant specific primer sequences can be found in S2B Fig.

Other qPCR primer sequences:

p21 Forward primer: AGCAGAGGAAGACCATGTGGA

p21 Reverse primer: GCGAGGCACAAGGGTACAA

SESN1 Forward: TACCTCAATGCTTAGACGGGCA

SESN1 Reverse: TCAGGAGTGCAAACAACAGTTT

BTG2 Forward primer: CTCCAGGAGGCACTCACAG

BTG2 Reverse primer: ATGATGGGGTCCATCTTGTG

ADCK3 Forward: TGATGCCTTTATCAACCCCCA

ADCK3 Reverse: CGAAGTATTCCAACTTGTCCCG

ANKRA2 Forward: TCACCCATAAAACAGTCAACCA

ANKRA2 Reverse: GCCAACTGGTGAACAGACAA

HSPA4L Forward: TTCTGCTTAGCGACTTGGGG

HSPA4L Reverse: GCTGCTGGTACTGAACCCTT

FASN Forward: GCTCCAGCCTCGCTCTC

FASN Reverse: TCTCCGACTCTGGCAGCTT

SCD Forward: TTCCTACCTGCAAGTTCTACACC

SCD Reverse: CCGAGCTTTGTAAGAGCGGT

SREBF1 Forward: GCCCCTGTAACGACCACTG

SREBF1 Reverse: CAGCGAGTCTGCCTTGATG

βActin forward primer: TTCAACACCCCAGCCATGT

βActin Reverse primer: GCCAGTGGTACGGCCAGA

### Microarray, ChIP and ChIP-seq

Gene expression microarray experiments were carried out on Illumina Human WG-6 version 2 arrays as described previously, using three biological replicates per condition [54]. ChIP and library preparation were performed as described previously [54]. In short, the immunoprecipitated DNA was end-repaired, A-tailed, ligated to the sequencing adapters, amplified by 18 cycles of PCR and size selected (200–300 bp) followed by single end sequencing on an Illumina Genome Analyzer IIx (GAIIx) according to the manufacturer’s recommendation. Antibodies used were: p53 (DO-1 Sigma); H3K4me3 (CMA304), H3K36me3 (CMA333) [54]. Expression microarray and ChIP-seq data are available at the National Center for Biotechnology Information Gene Expression Omnibus under accession numbers GSE53491 and GSE53379.

### Microarray analysis

All data analyses were carried out on R using Bioconductor packages [60]. Raw intensity data from the array scanner were processed using the BASH and HULK algorithms as implemented in the beadarray package [61,62]. Log2 transformation and quantile normalization of the data were performed across all sample groups. Differential expression analysis was carried out using the limma package [63]. Differentially expressed genes were determined by computing the log2 contrast between sh-p53#1 and vector control for each condition. Genes were selected using a p-value cut-off of <0.01 after application of FDR correction for multiple testing (Benjamini-Hochberg) applied globally to correct for multiple contrasts. Data were analysed through the use of IPA (Ingenuity® Systems, www.ingenuity.com/), and pathway enrichment was determined for genes with log2 ratio >0.58 or <-0.58 and an FDR corrected p-value < 0.01. Illumina HG6 v2 platform probe list was used as the background set. Pathway heatmaps were generated by plotting negative log of the Fisher’s exact test enrichment p-value against all pathways. The conditions were clustered by hierarchical clustering using R (R-project).

### ChIP-seq analyses

Single-end 36 bp reads generated by the Illumina GAIIx or High Seq were aligned against the Human Reference Genome (assembly hg18, NCBI Build 36) using BWA version 0.5.5. Reads were filtered by removing those with a BWA alignment quality score less than 15. A further filtration was carried out by removing reads falling into the ‘blacklist’ regions identified by ENCODE [64]. Principle Component Analysis (PCA) was used to assess the prevalence and quality of read data in TSS regions. Counts were normalized between samples by dividing by effective library size (bin count sums). The MACS algorithm version 1.4.1 was used together with hg18 aligned, sequence read BAM files for identifying peak regions representing p53 binding sites [65]. Peaks were inspected using the IGV Genome Browser (v 2.3) [66]. UCSC defined CpG islands (CGIs) were used to identify CGI overlapping peaks. Any peak that overlapped with a CGI was included in the CpG peakset and the remainder included in the non-CGI set. Peaks were mapped to genes using the ChIPpeakAnno BioConductor package and the EBI Peak Annotator. Ensembl 54 (hg18) gtf file downloaded from http://www.ensembl.org/info/data/ftp/index.html was used to annotate genes. The following region definitions were used when calculating genomic distribution of peaks: Core promoter regions (-3000 to +2000 around TSS), distal (-3000 to-50000), intergenic (> -50000) and downstream extremities (-2000 to +3000) around transcription end site.

pApo, acDDR and RIS samples had three biological replicates each, while the growing condition had two replicates. We identified a high confidence (HC) peak set consisting of replicated peaks. Peaks that were present in two or more replicates in each condition were included in the HC peak set. Non-replicated singleton peaks in each condition was then compared to peaks in other conditions. Peaks in one condition overlapping with at least two other conditions or peaks in one condition overlapping with peaks in at least two replicates from another condition were also included in the HC peak set. Finally all overlapping peaks were merged to get the final high confidence peak set for each condition.

We downloaded the FastQ files for the public data sets and aligned them to hg18 reference genome using BWA and removed contaminants using FastQC [67]. BAM files were generated and peak calling was performed using MACS v1.4.1. All other analysis was performed as described.

The analysis of gene annotation enrichment was performed using GREAT (http://great.stanford.edu/) using the ‘basal plus extension’ association rules with proximal 10kb upstream and 5kb downstream regulatory domain settings, and the whole human genome (hg18) as background [68].

### Rcade

Peak distributions were plotted and bins 50 bp upstream and 1500 bp downstream of TSS were defined based on p53 signal enrichment. baySeq was used to determine enrichment over input [69]. Counts were normalized using the Quantile method (baySeq package). ChIP-seq and expression data were combined using a Bayesian approach, ranking genes in order of probability of being a p53 target. For each probe, we calculate the posterior probability of a p53 effect on transcription, Pr (DE and C | data), as proportional to Pr(DE | data) Pr(C | data)—here, Pr(DE | data) is the limma-derived posterior probability of differential expression under p53 knockdown, Pr(C | data) is the baySeq-derived posterior probability of enrichment for ChIP. Each probe’s posterior probability was logit transformed into a B value, through applying the logit transformation. Probes with B value greater than the threshold-1.5 were taken forward in the analysis. IPA upstream regulator analysis method was used as a sequence independent method to confirm the transcriptional regulators of the Rcade gene lists. The DAVID bioinformatics resource (v6.7) was used for ontology enrichment analysis of Rcade genes. Illumina HG6 v2 platform probe list was used as the background probe set [70].

### Phenotype specific knowledge based Pathway modeling

Biochemical models of the p53 regulome (the set of p53 regulated genes) for each phenotype under consideration was constructed utilizing the following integrative and iterative analytical approach. Putative p53 targets were identified by integrating ChIP-seq and expression datasets using the Rcade method (Bioconductor). Rcade genes with a B value >-1.5 were selected as putative p53 targets for further analysis. Reviews, primary scientific publications and phenotype associated biochemical pathways and signaling, regulatory, metabolic and physical interactions involved in each of the conditions were used to build a phenotype specific global network framework. The selected Rcade genes were then used to extract pathway information from multiple public (KEGG, Reactome, Wikipathways, Pathway Commons, Panther etc.) and commercial pathway (Ingenuity Pathway Analysis) databases [71–75]. Pathways involved were integrated into the model in combination with information integrated from interaction databases and ontology analysis followed by extensive semi-automated literature mining. Sub-cellular localization information and p53 related protein-protein or genetic interactions were integrated by mining relevent biological databases (InAct, Biogrid, String, IPA, MitoCarta) [76–79]. Regulation of p53 or by p53 and interaction with p53 or evidence of contribution to or involvement in phenotype for each interaction was documented. Expert manual curation was used to build and iteratively refine these detailed biochemical models of p53 targets. Nodes are represented by p53 induced, repressed genes and those not regulated by p53 providing pathway context. Edges are represented by color-coded arrows denoting catalytic, protein-protein, inhibitory, direct functional, translocation or undefined interactions. A large number of p53 regulated genes identified as p53 interacting or p53 stability modifying proteins documented in S4 and S5 Fig. are shown in detail as regulatory p53 hubs in Fig. 4A. A list of p53 hub genes and evidence for p53 association are provided in S6 Table.

### Functional association networks

The Multiple Association Network Integration Algorithm was used to identify functional association networks. The method uses a large dataset of over 300 functional association networks that are grouped into five categories: co-localization, genetic interaction, physical interaction, predicted interactions and shared protein domains. Networks are weighted according to source-dependent criteria, stored as sparse weighted adjacency matrices, where weight corresponds to gene interaction strength. The algorithm uses the Rcade list to integrate association networks from multiple sources into a composite network using a conjugate gradient optimization method. The computation consists of two parts; an algorithm, based on linear regression, for calculating a single composite functional association network from multiple networks derived from different genomic and proteomic datasets; and a Gaussian label propagation algorithm for predicting gene function given this composite network. Strength of the functional relatedness is represented by the edge density. Network topology and connectivity analysis and biological enrichment analysis of the inferred network was carried out. To determine the specificity of the method we used a similar sized set of random genes (derived from the universe of human protein coding genes) and the above network inference methods were applied. This resulted in an extremely sparse network, in which the majority of nodes remained unconnected. The exclusion or inclusion of p53 within the random list had no effect on its connectivity. Networks analysis and visualization was performed with Cytoscape (ver 2.8.3) software [80].

### p53 interactome analysis

Biogrid database (ver 3.2) was programmatically accessed by perl scripts using the RESTful API. The database was queried with a list of (pro-apoptosis or senescence) putative p53 target genes (Rcade genes with a B value > -1.5). Protein-protein interactions were filtered by Rcade lists and then by those consisting of either of the interacting partners being on the previously identified p53 hub gene lists. Interactions between hub genes were clustered into those between p53, MDM2 and other hub genes and visualized as a circos plot using the Circos program (ver 0.64) [81].

### Motif analyses

*CDKN1A* and *SCD* sequences in fasta format were used for transcription factor binding site analysis. The TransfacPro (v 2013.2) MATCH algorithm, together with transcription factor position weight matrices and specificity profiles was used to identify TP53 and other transcription factor binding sites [82]. We used the minSUM_good profile to restrict analysis to only high quality matrices and to minimize the sum of both false positive and negative error rates. *De novo* motif enrichment analysis was performed using MemeChIP package [83] and Position Weight Matrix (PWM) scanning based motif enrichment analysis was performed using PscanChip with TransfacPro PWMs and open chromatin background downloaded from UCSC genome browser [84–86]. Distribution of p53 motifs were defined by CentDist [87].

### Recursive partitioning analysis

Recursive partitioning (RP) was carried out using the R *party* package on normalized gene expression data from the four datasets, all derived from Affymetrix array platforms [31–33]. Genes were selected as having expression profiles that could stratify patients into subgroups with significantly different survival outcomes, by selecting those genes for which the most significant stratification had a p-value (adjusted for multiple correction) of <0.05. p53 expression and mutation status was derived from CEL files for the Miller et al. [38] dataset were downloaded from GEO (Accession number: GSE3494). All data analyses were carried out on R using Bioconductor packages. The data were normalized using the RMA algorithm. Differential expression analysis was carried out using the *limma* package.

### TFBS enrichment analysis of putative p53 targets

p53 bound, transcriptionally active, putative p53-target genes were derived from Rcade analysis of pro-apoptotic and oncogene RAS-induced senescence conditions. Genes with an Rcade B-value > -1.5 was used for further analysis. Core promoter sequences (-3kb and +2kb around the TSS) were extracted and transcription factor binding site (TFBS) enrichment analysis was performed using the Pscan program together with vertebrate TFBS position weight matrices from the Transfac professional database (v2013.4) [85,88]. Promoter sequences of equal length to the test set from all protein coding genes were used as a background set.

## Supporting Information

S1 Fig Phenotype associated p53 activation. **(A)** E1A/RAS expressing (pro-apoptotic, pApo) cells are sensitive to DNA damage-induced cell death, compared to growing (Grow) and RAS-induced senescent (RIS) cells. Cells were treated with 0.5 μg/ml Doxorubicin (DOXO) or DMSO (No) for 24 hours, and cell viability was assessed by trypan blue exclusion (mean ± SEM; n ≥ 3). ***p < 0.001. Representative phase contrast images of pApo cells are also shown. **(B)** qPCR analysis of known p53-targets in each condition (mean ± SEM; n = 3) with or without sh-p53#1. **(C)** qPCR analysis of known p53-targets as well as p53 in each condition (mean ± SEM; n = 3) with or without sh-p53#2. **(D)** Pathway heatmaps for genes down-regulated (positively regulated by p53) and up-regulated (negatively regulated by p53) upon p53 depletion (sh-p53#1). Grow, growing IMR90 cells; acDDR, cells treated with 100 μM etoposide for 24h; RIS, RAS-induced senescent cells; pApo, E1A/RAS expressing pro-apoptotic cells.
(TIF)

S2 Fig p53 CGI peak in *CDKN1A* (*p21*) locus and its transcripts. **(A)** Sequence of the proximal CGI in the *p21* locus and potential p53 binding motifs (highlighted) defined using TransfacPro Match algorithm and associated matrices with 0.8 cut-offs for both core and matrix scores. **(B)** Two representative *p21* Refseq transcripts, variant 1 (the classic transcript) and variant 2. RT-qPCR primer sets were designed to amplify each variant separately or both variants simultaneously. **(C)** RT-qPCR analyses of the *p21* transcripts in cells in the indicated conditions (mean ± SEM; n = 3). Both variants were up-regulated in all conditions.
(TIF)

S3 Fig Integration of p53-dependent expression and p53 ChIP-seq data. **(A)** Venn diagrams showing the numbers of HC p53-ChIP-seq peaks in the indicated conditions genome-wide or within the promoter regions. **(B)** Dot plots showing p53 binding intensity against differential expression (DE). Each point represents a gene, with its associated DE log-ratio plotted on the x-axis and the bin-derived ChIP log-ratio on the y-axis. The color of each point represents the log-odds that Rcade has assigned to that gene: that is, how likely that gene is to be a direct p53 target. We can see the genes that are likely to be p53 targets at the top-left and top-right of the graphs. **(C)** Venn diagram showing the numbers of Rcade-derived p53-targets in both chronic conditions. Overlap is smaller compared to the HC peak sets—see the right Venn diagram in (A). **(D)** DAVID analysis for Rcade-derived genes in each condition. Red script represents processes associated with typical p53-related functions such as cell cycle, DNA damage response, and apoptosis; blue script represents processes associated with RNA metabolism and regulation; green script represents processes associated with membrane-bound organelles. DAVID ontologies were manually trimmed by removing apparent redundancy. **(E)** qPCR showing fold change of indicated mRNAs after p53 knockdown in the indicated conditions (mean ± SEM; n = 3). **(F)** qPCR showing fold change of indicated mRNAs after p53 induction in tetracycline-inducible p53 expressing H1299 cells (mean ± SEM; n = 3). Inset is immunoblot analysis of p53 in the presence or absence of 100μM doxycycline (Dox) for 24h. *p < 0.05, **p < 0.01, ***p < 0.001.
(TIF)

S4 Fig Phenotype specific knowledge based pathway models of putative p53 targets in pro-apoptotic condition. Maroon ovals indicate positive and blue ovals indicate negative regulation by p53 (red oval). White ovals are not regulated by p53 but are involved in the pathways. The blue T-lines show inhibition, green lines ending in a circle are enzymatic reactions, orange arrows are protein-protein interactions, pink dotted lines are either translocation or degradation, black lines are undefined interactions and black dotted lines are indirect interactions. Numbers associated with genes or connections represent are linked to pubmed IDs providing the evidence for the interaction. A high-resolution version of the figure is available at http://australian-systemsbiology.org/tp53.
(TIF)

S5 Fig Phenotype specific knowledge based pathway models of putative p53 targets in RIS condition. Maroon ovals indicate positive and blue ovals indicate negative regulation by p53 (red oval). White ovals are not regulated by p53 but are involved in the pathways. The blue T-lines show inhibition, green lines ending in a circle are enzymatic reactions, orange arrows are protein-protein interactions, pink dotted lines are either translocation or degradation, black lines are undefined interactions and black dotted lines are indirect interactions. Numbers associated with genes or connections represent are linked to pubmed IDs providing the evidence for the interaction. A high-resolution version of the figure is available at http://australian-systemsbiology.org/tp53.
(TIF)

S6 Fig Heatmap summarizing recursive partitioning analysis for Rcade-derived p53 targets in the pApo condition for four cancer data sets as in Fig. 5A. *MDM2*, *CDKN1A* (green) and *SCD* (red) are highlighted. Consistent with the recent study by Kenzelmann Broz et al. [13], higher levels of genes involved in autophagy (brown) were often associated with better prognosis.
(TIF)

S7 Fig SCD is a p53-repressive target. **(A)** Immunoblot analysis in p53-inducible H1299 cells at d3 after different doses of doxycycline addition. 293T cells express a high level of ‘inactive’ p53 due to the expression of SV40 Large T antigen. **(B)** The sequence of the p53-bound region in the SCD promoter as shown in Fig. 6F. Potential p53 motifs are highlighted as in S2A Fig This region was used for the reporter assay in Fig. 6G. **(C)** qPCR showing fold change of indicated mRNAs in each condition (mean ± SEM; n = 3) with or without sh-p53#2. **(D)** qPCR showing fold change of indicated mRNAs after p53 induction in tetracycline-inducible p53 expressing H1299 cells as in S3F Fig (mean ± SEM; n = 3). Dox+, 100 μM doxycycline treatment for 24h. **p < 0.01, ***p < 0.001. **(E)** Immunoblot analysis showing that SREBF1 is not down-regulated during RIS in IMR90 cells. The assay was performed at d10 post selection for RAS as in Fig. 6B.
(TIF)

S1 Table Microarray: Differentially expressed (DE) genes upon p53 knockdown (sh-p53#1). Core genes (intersection of DE genes from RIS, pApo, and acDDR conditions in Fig. 1F) are shown.
(XLSX)

S2 Table ChIP-Seq replicate information (number of replicates, reads etc.) (XLSX)

S3 Table Genomic distribution of subsets of high confidence p53 ChIP-seq peaks in each condition. Genomic features were determined as in Fig. 2B. Same numbers of p53 high confidence peaks were selected based on MACS score from each condition.
(XLS)

S4 Table GO analysis for genes associated with CGI- and non-CGI-p53 peaks in each condition. Blue script represents processes associated with RNA metabolism and regulation, whereas red script represents processes associated with typical p53 related functions such as cell cycle, DNA damage response, and apoptosis.
(XLSX)

S5 Table Rcade-derived genes in RIS and pApo conditions. Genes with Rcade B-value greater than the threshold-1.5 are included.
(XLSX)

S6 Table p53 Hub genes shown in Fig. 4 in RIS and pApo conditions. Evidence for interactions are provided as Pubmed IDs (PMID).
(XLSX)

S7 Table Enrichment of known transcription factor binding sites (TFBS) in the Rcade gene set. Only transcription factors from a non-redundant vertebrate PWM set with enrichment p-value > 0.01 are shown.
(XLSX)
