# Supplementary figures and images for: Phenotype Specific Analyses Reveal Distinct Regulatory Mechanism for Chronically Activated p53
Source: PLoS Genet. 2015 Mar 19;11(3):e1005053. doi: 10.1371/journal.pgen.1005053 (PMC4366240; doi:10.1371/journal.pgen.1005053)

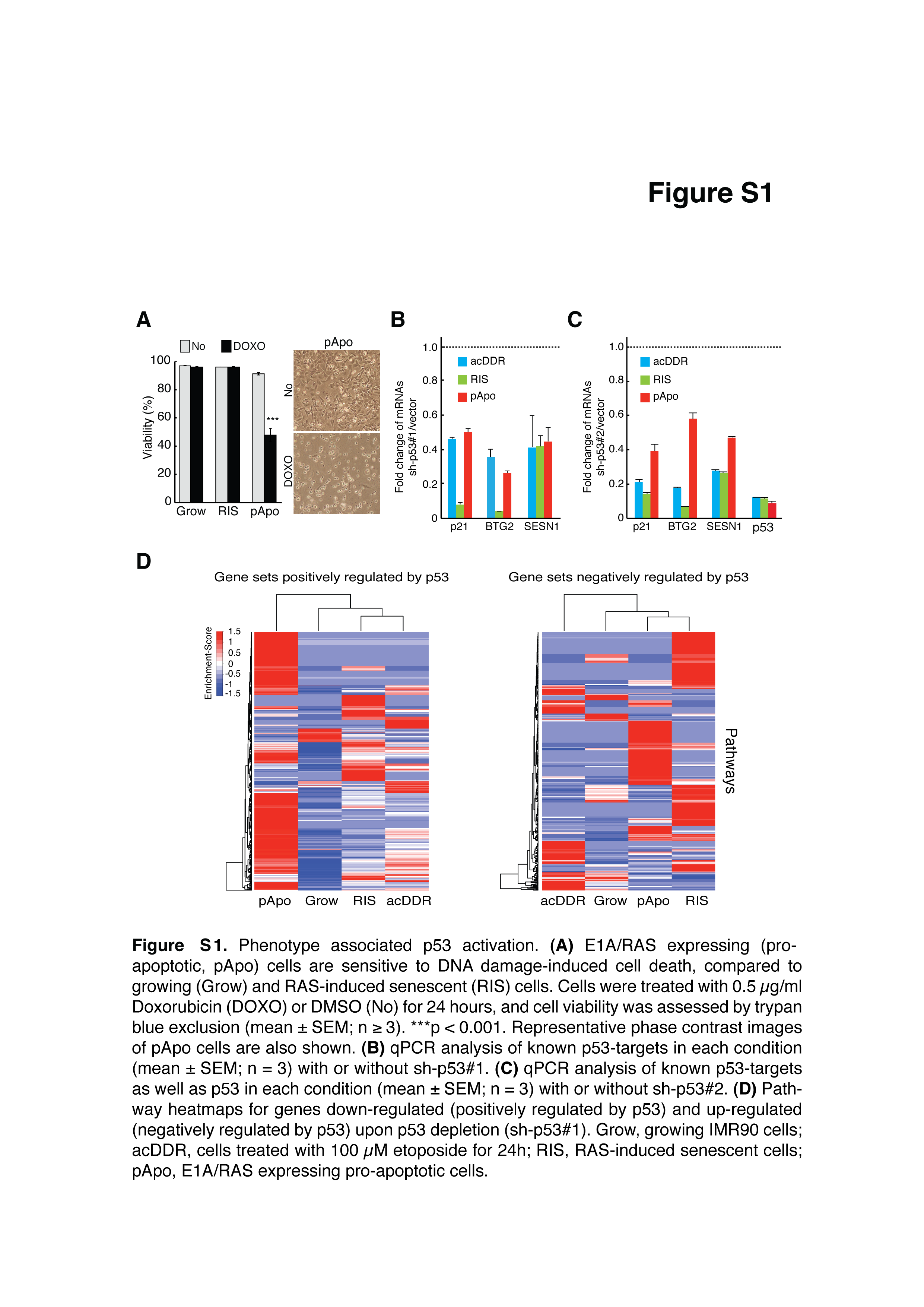

Supplement: S1 Fig — (A) E1A/RAS expressing (pro-apoptotic, pApo) cells are sensitive to DNA damage-induced cell death, compared to growing (Grow) and RAS-induced senescent (RIS) cells. Cells were treated with 0.5 μg/ml Doxorubicin (DOXO) or DMSO (No) for 24 hours, and cell viability was assessed by trypan blue exclusion (mean ± SEM; n ≥ 3). ***p < 0.001. Representative phase contrast images of pApo cells are also shown. (B) qPCR analysis of known p53-targets in each condition (mean ± SEM; n = 3) with or without sh-p53#1. (C) qPCR analysis of known p53-targets as well as p53 in each condition (mean ± SEM; n = 3) with or without sh-p53#2. (D) Pathway heatmaps for genes down-regulated (positively regulated by p53) and up-regulated (negatively regulated by p53) upon p53 depletion (sh-p53#1). Grow, growing IMR90 cells; acDDR, cells treated with 100 μM etoposide for 24h; RIS, RAS-induced senescent cells; pApo, E1A/RAS expressing pro-apoptotic cells. (TIF) [file pgen.1005053.s001.tif]

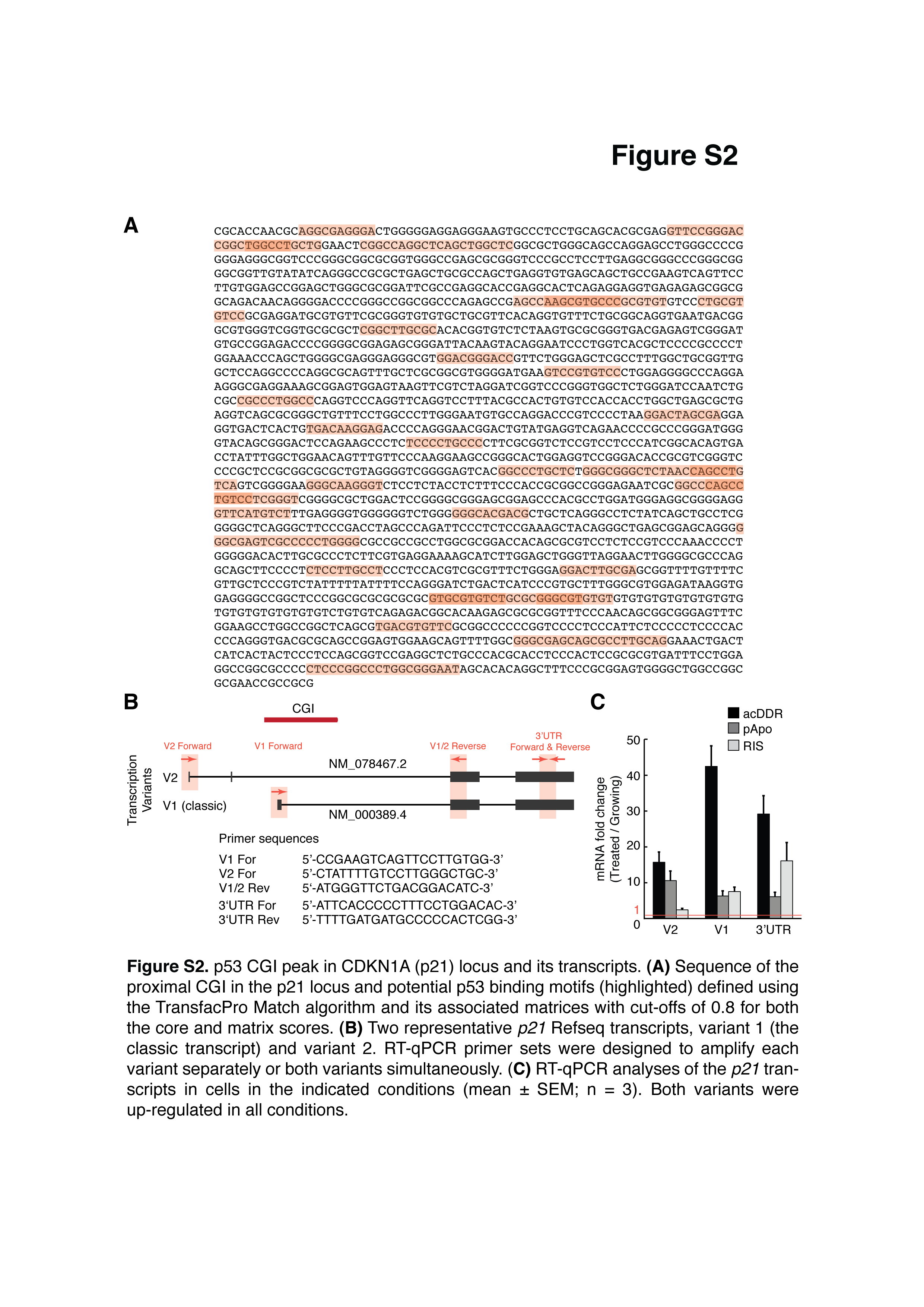

Supplement: S2 Fig — (A) Sequence of the proximal CGI in the p21 locus and potential p53 binding motifs (highlighted) defined using TransfacPro Match algorithm and associated matrices with 0.8 cut-offs for both core and matrix scores. (B) Two representative p21 Refseq transcripts, variant 1 (the classic transcript) and variant 2. RT-qPCR primer sets were designed to amplify each variant separately or both variants simultaneously. (C) RT-qPCR analyses of the p21 transcripts in cells in the indicated conditions (mean ± SEM; n = 3). Both variants were up-regulated in all conditions. (TIF) [file pgen.1005053.s002.tif]

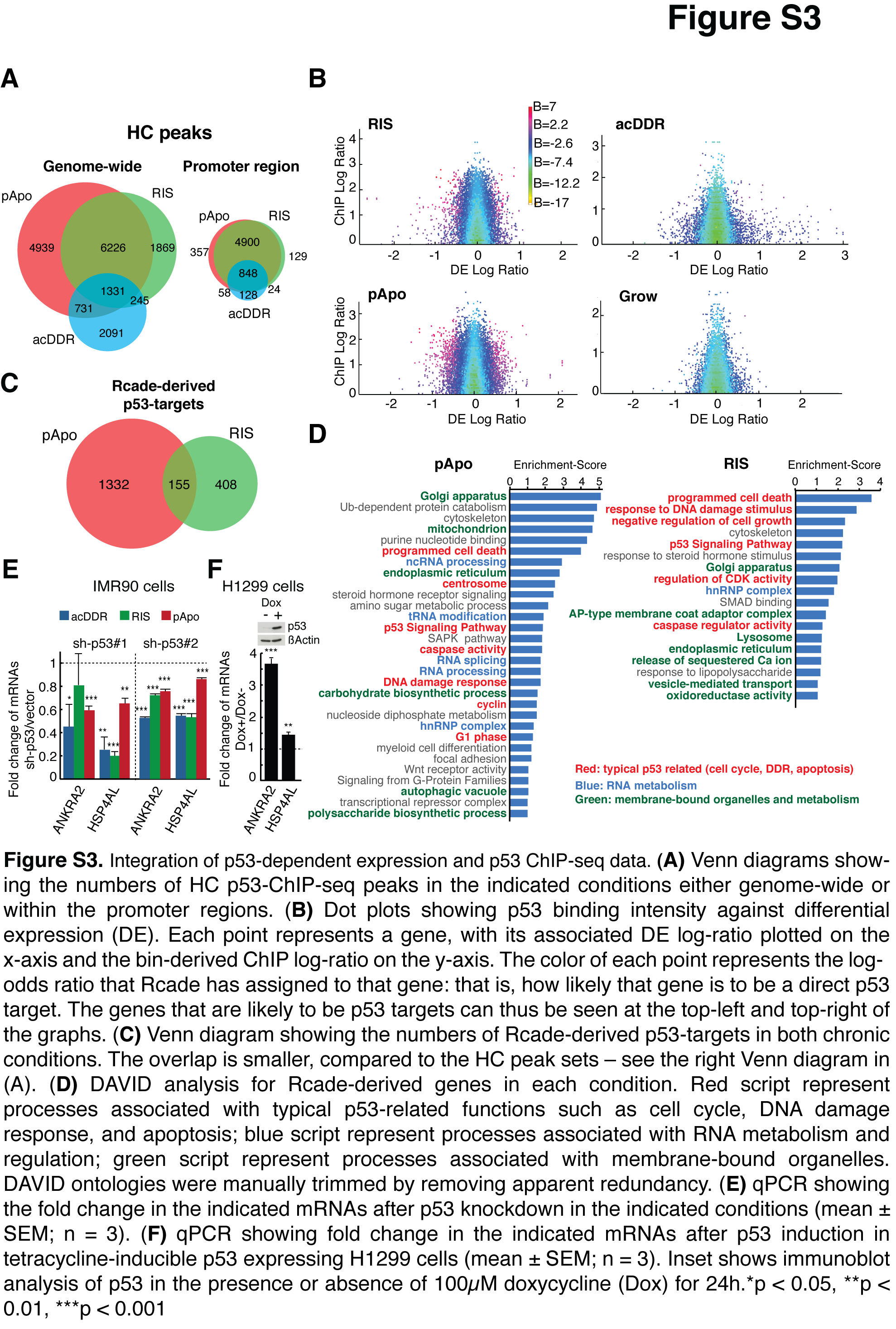

Supplement: S3 Fig — (A) Venn diagrams showing the numbers of HC p53-ChIP-seq peaks in the indicated conditions genome-wide or within the promoter regions. (B) Dot plots showing p53 binding intensity against differential expression (DE). Each point represents a gene, with its associated DE log-ratio plotted on the x-axis and the bin-derived ChIP log-ratio on the y-axis. The color of each point represents the log-odds that Rcade has assigned to that gene: that is, how likely that gene is to be a direct p53 target. We can see the genes that are likely to be p53 targets at the top-left and top-right of the graphs. (C) Venn diagram showing the numbers of Rcade-derived p53-targets in both chronic conditions. Overlap is smaller compared to the HC peak sets—see the right Venn diagram in (A). (D) DAVID analysis for Rcade-derived genes in each condition. Red script represents processes associated with typical p53-related functions such as cell cycle, DNA damage response, and apoptosis; blue script represents processes associated with RNA metabolism and regulation; green script represents processes associated with membrane-bound organelles. DAVID ontologies were manually trimmed by removing apparent redundancy. (E) qPCR showing fold change of indicated mRNAs after p53 knockdown in the indicated conditions (mean ± SEM; n = 3). (F) qPCR showing fold change of indicated mRNAs after p53 induction in tetracycline-inducible p53 expressing H1299 cells (mean ± SEM; n = 3). Inset is immunoblot analysis of p53 in the presence or absence of 100μM doxycycline (Dox) for 24h. *p < 0.05, **p < 0.01, ***p < 0.001. (TIF) [file pgen.1005053.s003.tif]

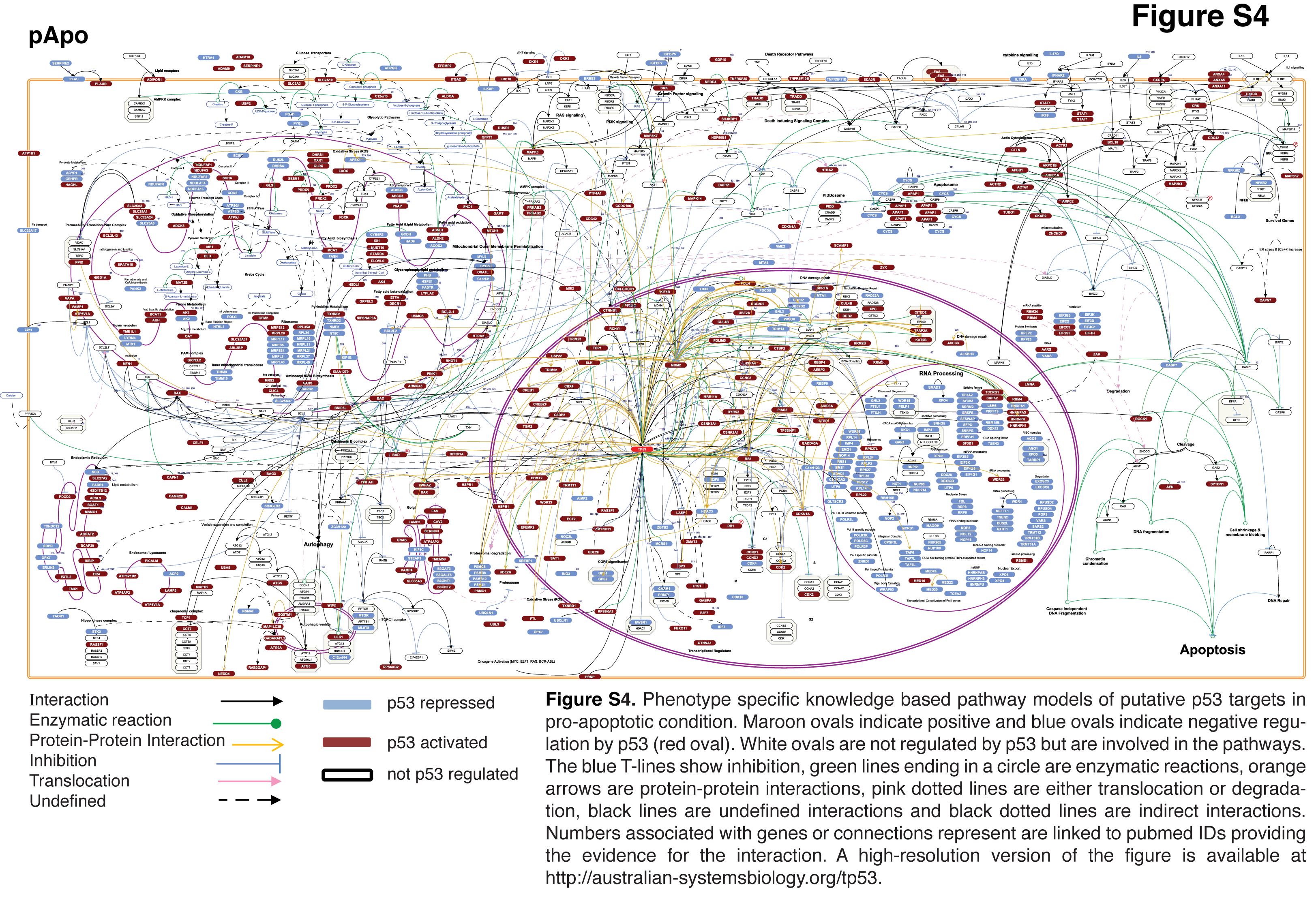

Supplement: S4 Fig — Maroon ovals indicate positive and blue ovals indicate negative regulation by p53 (red oval). White ovals are not regulated by p53 but are involved in the pathways. The blue T-lines show inhibition, green lines ending in a circle are enzymatic reactions, orange arrows are protein-protein interactions, pink dotted lines are either translocation or degradation, black lines are undefined interactions and black dotted lines are indirect interactions. Numbers associated with genes or connections represent are linked to pubmed IDs providing the evidence for the interaction. A high-resolution version of the figure is available at http://australian-systemsbiology.org/tp53. (TIF) [file pgen.1005053.s004.tif]

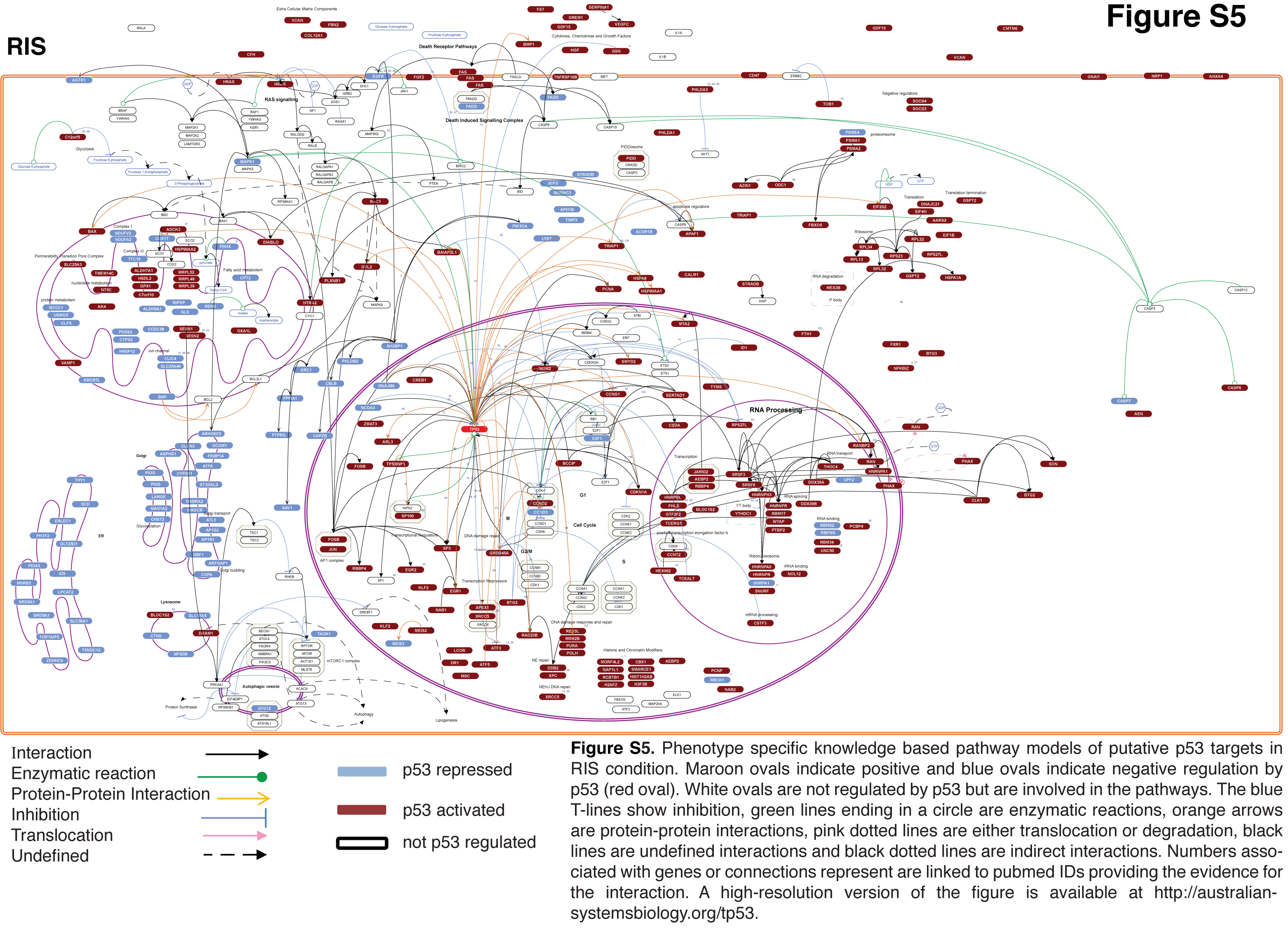

Supplement: S5 Fig — Maroon ovals indicate positive and blue ovals indicate negative regulation by p53 (red oval). White ovals are not regulated by p53 but are involved in the pathways. The blue T-lines show inhibition, green lines ending in a circle are enzymatic reactions, orange arrows are protein-protein interactions, pink dotted lines are either translocation or degradation, black lines are undefined interactions and black dotted lines are indirect interactions. Numbers associated with genes or connections represent are linked to pubmed IDs providing the evidence for the interaction. A high-resolution version of the figure is available at http://australian-systemsbiology.org/tp53. (TIF) [file pgen.1005053.s005.tif]

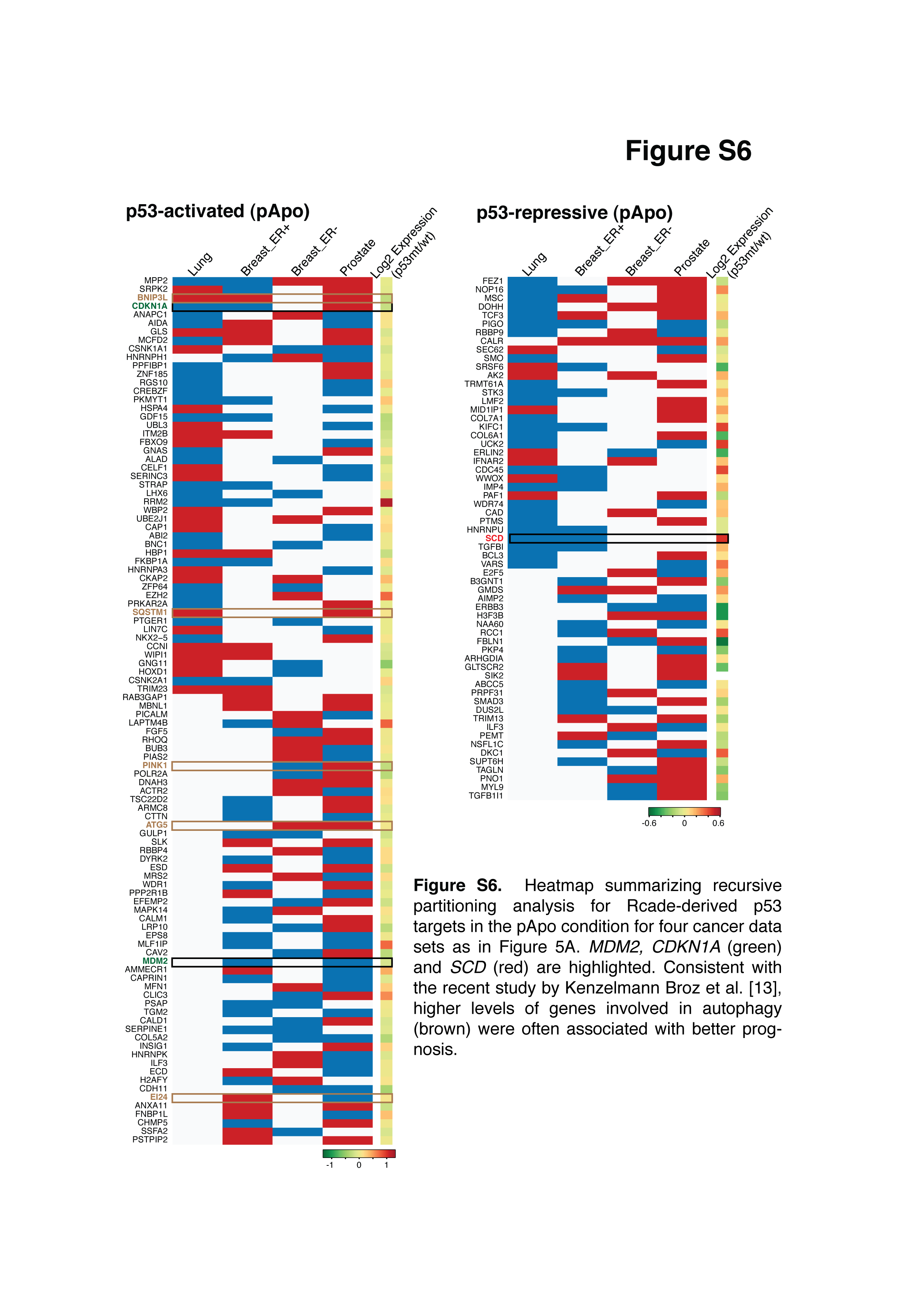

Supplement: S6 Fig — MDM2, CDKN1A (green) and SCD (red) are highlighted. Consistent with the recent study by Kenzelmann Broz et al. [13], higher levels of genes involved in autophagy (brown) were often associated with better prognosis. (TIF) [file pgen.1005053.s006.tif]

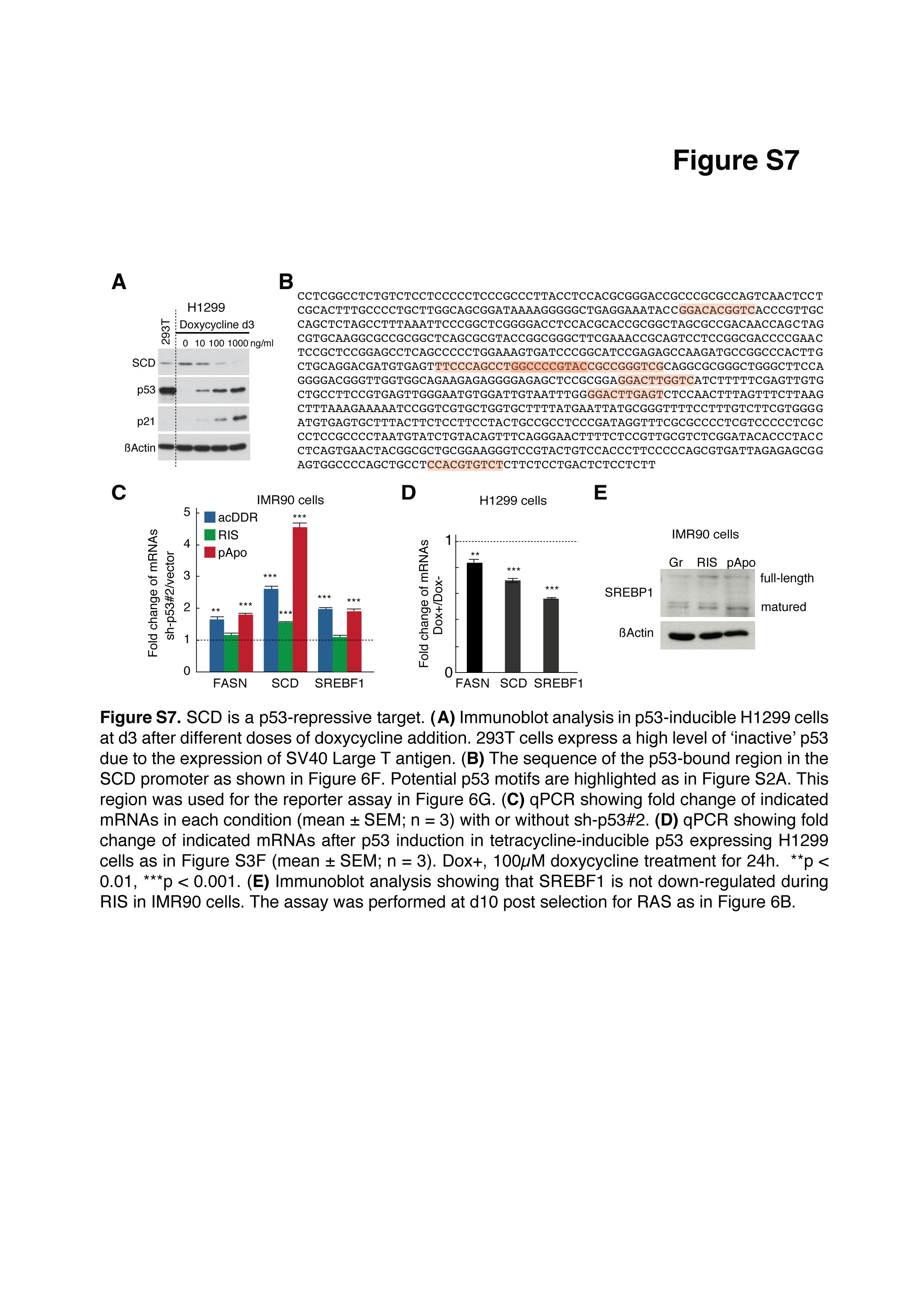

Supplement: S7 Fig — (A) Immunoblot analysis in p53-inducible H1299 cells at d3 after different doses of doxycycline addition. 293T cells express a high level of ‘inactive’ p53 due to the expression of SV40 Large T antigen. (B) The sequence of the p53-bound region in the SCD promoter as shown in Fig. 6F. Potential p53 motifs are highlighted as in S2A Fig This region was used for the reporter assay in Fig. 6G. (C) qPCR showing fold change of indicated mRNAs in each condition (mean ± SEM; n = 3) with or without sh-p53#2. (D) qPCR showing fold change of indicated mRNAs after p53 induction in tetracycline-inducible p53 expressing H1299 cells as in S3F Fig (mean ± SEM; n = 3). Dox+, 100 μM doxycycline treatment for 24h. **p < 0.01, ***p < 0.001. (E) Immunoblot analysis showing that SREBF1 is not down-regulated during RIS in IMR90 cells. The assay was performed at d10 post selection for RAS as in Fig. 6B. (TIF) [file pgen.1005053.s007.tif]
